# Understanding the Genetic Diversity of *Mycobacterium africanum* Using Phylogenetics and Population Genomics Approaches

**DOI:** 10.3389/fgene.2022.800083

**Published:** 2022-04-13

**Authors:** Muthukumar Balamurugan, Ruma Banerjee, Sunitha Manjari Kasibhatla, Archana Achalere, Rajendra Joshi

**Affiliations:** HPC—Medical and Bioinformatics Applications Group, Centre for Development of Advanced Computing, Innovation Park, Pune, India

**Keywords:** *Mycobacterium africanum*, population genomics, SNP, lineage, bioinformatics

## Abstract

A total of two lineages of *Mycobacterium tuberculosis* var. *africanum* (*Maf*), L5 and L6, which are members of the *Mycobacterium tuberculosis* complex (MTBC), are responsible for causing tuberculosis in West Africa. Regions of difference (RDs) are usually used for delineation of MTBC. With increased data availability, single nucleotide polymorphisms (SNPs) promise to provide better resolution. Publicly available 380 *Maf* samples were analyzed for identification of *“core-cluster-specific-SNPs,”* while additional 270 samples were used for validation. RD-based methods were used for lineage-assignment, wherein 31 samples remained unidentified. The genetic diversity of *Maf* was estimated based on genome-wide SNPs using phylogeny and population genomics approaches. Lineage-based clustering (L5 and L6) was observed in the whole genome phylogeny with distinct sub-clusters. Population stratification using both model-based and *de novo* approaches supported the same observations. L6 was further delineated into three sub-lineages (L6.1–L6.3), whereas L5 was grouped as L5.1 and L5.2 based on the occurrence of RD711. L5.1 and L5.2 were further divided into two (L5.1.1 and L5.1.2) and four (L5.2.1–L5.2.4) sub-clusters, respectively. Unassigned samples could be assigned to definite lineages/sub-lineages based on clustering observed in phylogeny along with high-confidence posterior membership scores obtained during population stratification. Based on the (sub)-clusters delineated, “*core-cluster-specific-SNPs*” were derived. Synonymous SNPs (137 in L5 and 128 in L6) were identified as biomarkers and used for validation. Few of the cluster-specific missense variants in L5 and L6 belong to the central carbohydrate metabolism pathway which include His6Tyr (Rv0946c), Glu255Ala (Rv1131), Ala309Gly (Rv2454c), Val425Ala and Ser112Ala (Rv1127c), Gly198Ala (Rv3293) and Ile137Val (Rv0363c), Thr421Ala (Rv0896), Arg442His (Rv1248c), Thr218Ile (Rv1122), and Ser381Leu (Rv1449c), hinting at the differential growth attenuation. Genes harboring multiple (sub)-lineage-specific *“core-cluster”* SNPs such as Lys117Asn, Val447Met, and Ala455Val (Rv0066c; *icd2*) present across L6, L6.1, and L5, respectively, hinting at the association of these SNPs with selective advantage or host-adaptation. Cluster-specific SNPs serve as additional markers along with RD-regions for *Maf* delineation. The identified SNPs have the potential to provide insights into the genotype–phenotype correlation and clues for endemicity of *Maf* in the African population.

## 1 Introduction

The genus *Mycobacterium* is known to cause tuberculosis (TB), which infects ∼10 million people worldwide annually (Coscolla and Gagneux 2014; Gagneux 2018; Global Tuberculosis Report 2021). The disease burden associated with TB is enormous, and Africa is one of the severely affected continents (Gehre et al., 2016; Global Tuberculosis Report 2021). Human TB is caused mainly by the organism *Mycobacterium tuberculosis* (*M. tuberculosis*), which belongs to the *Mycobacterium tuberculosis* complex (MTBC) (Gagneux 2018; Kanabalan et al., 2021). MTBC is responsible for TB in humans and animals (Brosch et al., 2002; Gagneux 2018). MTBC lineages have undergone specific deletion of large sequences in their genomes, known as the region of difference (RD), which enables delineation (Brosch et al., 2002). Lineage-wise classification of MTBC is also enabled using restriction fragment length polymorphism (RFLP) and PCR, such as mycobacterial interspersed repetitive units-variable number of tandem repeats (MIRU-VNTR) spoligotyping (Jeon et al., 2018).

There are seven lineages of MTBC, of which lineages L1–L4 and L7 comprise the *Mycobacterium tuberculosis* sensu stricto (majorly infecting humans)*,* and L5 and L6 consist of *Mycobacterium tuberculosis* var. *africanum* (hereafter referred to as *Maf*) (Blouin et al., 2012; Firdessa et al., 2013; Riojas et al., 2018; Gagneux 2018). Additional lineages include two recently identified lineages, namely, L8 and L9 (Ngabonziza et al., 2020; Coscolla et al., 2021) and other animal-adapted strains (Gagneux 2018). According to RD-based classification, L5 and L6 evolved from their most recent ancestor that underwent RD9 deletion (Gagneux 2018). Besides RD9 deletion, L5 has also undergone RD711 deletion, and L6 has undergone RD7, RD8, RD10 (as also seen in the animal-adapted strains of MTBC), and RD702 deletion (de Jong et al., 2010b). Phylogenetically, this places L5 closer to the human-adapted MTBC and L6 closer to the animal-adapted strains (Gagneux 2018). Recent studies have further classified L5 based on the presence and absence of RD711 (Ates et al., 2018; Coscolla et al., 2021; Comín et al., 2021).

Circulation of all MTBC lineages has been reported in Africa, thereby suggesting the emergence of MTBC from a common ancestor in Africa and its spread and expansion to the rest of the world through human migration (Gagneux et al., 2006, Wirth et al., 2008, Comas et al., 2013; Gehre et al., 2016, Rutaihwa et al., 2019, O’Neill et al., 2019; Coscolla et al., 2021). Among the human-associated MTBC, most of the lineages are found to be geographically widespread. *Maf* (L5 and L6) is restricted, particularly to the western region of West Africa and is known to cause 40–50% of TB in West Africa (Chatterjee and Pramanik 2015; Gehre et al., 2016; Winglee et al., 2016; Baya et al., 2020). Conversely, L7–L9 are limited to East Africa (Blouin et al., 2012; Firdessa et al., 2013; Ngabonziza et al., 2020; Coscolla et al., 2021). The geographical restriction of *Maf* infection is still elusive; however, few studies have reported the occurrence of TB due to *Maf* in other parts of the world, mostly in individuals who have migrated from the endemic parts of Africa (Isea-Peña et al., 2012; Comín et al., 2021). Such pathogens having adaptation to infect specific hosts restricted to a particular geographical location are termed as “specialists,” and this behavior may be attributed to the strict host–pathogen interactions which are relatively understudied (Brites and Gagneux, 2015; Asante-Poku et al., 2016; Sriswasdi et al., 2017).

L5 and L6 are known to differ substantially from other MTBC members in terms of genetic diversity, growth, and metabolism (Yeboah-Manu et al., 2017). Compared to *M.tuberculosis*, L5 and L6 are reported to have an attenuated and slower growth in culture along with lower bacterial load and delayed disease progression (Cá et al., 2019; Baya et al., 2020). L6 is known to be an opportunistic pathogen owing to mutations in genes essential for growth and contributes toward latent TB burden in West Africa (de Jong et al., 2005; de Jong et al., 2010a; Gehre et al., 2013; Ofori-Anyinam et al., 2017). Similar studies for L5 genomics are limited that has been highlighted earlier (Yeboah-Manu et al., 2017; Ates et al., 2018; Coscolla et al., 2021; Sanoussi et al., 2021). A slower response to TB treatment for L6 was observed when compared to other *sensu stricto* lineages (Diarra et al., 2018). Identification and treatment of latent TB are essential for reducing deaths caused by TB, as emphasized by the “End TB Strategy” of the World Health Organization (WHO) (Uplekar and Raviglione 2015; WHO 2015a, WHO 2015b; Zellweger et al., 2020). There is a lot of interest to understand the variation of *Maf* with respect to epidemiology and virulence (Asante-Poku et al., 2016; Stucki et al., 2016; Yeboah-Manu et al., 2017; Gagneux 2018; Ates et al., 2018; Coscolla et al., 2021). Phylogenomic distribution studies of MTBC lineages using comparative genomics approaches are studied extensively (Brosch et al., 2002, Gagneux et al., 2006; Vasconcellos et al., 2010, Gehre et al., 2016; Gagneux 2018; Coscolla et al., 2021).

Apart from long sequence polymorphisms, such as RDs and tandem repeats, signature genome-wide single nucleotide polymorphism (SNP)-based stratification approaches promise to provide valuable insights into the genomic diversity and help delineate the epidemiology of the circulating strains (Lipworth et al., 2019; Napier et al., 2020).

Population stratification studies based on genome-wide SNPs enable unraveling the genetic diversity existing within bacterial populations (Takuno et al., 2012; Lee et al., 2015; Castillo et al., 2020). Earlier studies by Lee et al. (2015) using *M.tuberculosis* pertaining to a specific geographical location provided insight into the role of evolutionary forces that shape the pathogen evolution *vis-a-vis* its environment. Hence, understanding population stratification would aid in rapid identification of (sub)-lineages, which is of great significance in tuberculosis research and may help in understanding the origin and predicting future outbreaks through the identification of rapid diagnostic markers (MacLean et al., 2019; Singh et al., 2019). To gain an insight into the population genetic characteristic of *Maf* samples (L5 and L6), an integrative approach using *de novo* and model-based clustering methods along with different population genomics approaches have been carried out based on the genome-wide variant profile generated with reference to *Mycobacterium tuberculosis* H37Rv. The variants identified may also have the potential to serve as robust genetic markers for differentiation of lineages and sub-lineages and provide clues towards host adaptation of *Maf*.

## 2 Materials and Methods

### 2.1 Data Collection and Processing

Sequence data of whole-genome belonging to 572 *Maf* samples were downloaded from NCBI Sequence Read Archive (SRA) available as of December 2019, with the keyword-based search “*Mycobacterium africanum.*” Samples were collected from multiple SRA projects. Quality check was carried out for each sample using FastQC (Andrews, 2010). Read quality >28 were retained, and poor quality reads were trimmed by TrimGalore (Babraham Bioinformatics, 2019). Reference mapping for all *Maf* samples was carried out using BWA-MEM (version 0.7.17) with *Mycobacterium tuberculosis* H37Rv (Refseq id: NC_000962.3) genome as reference (Cole et al., 1998; Li 2013). Samples with less than a million reads were further filtered based on read depth (minimum DP 5X) and mapping quality (MQ > 40) criteria (Supplementary Table S1).

SAMtools/BCFtools were used for sorting, indexing, and merging of samples (Li 2011). Lineage identification was performed using a RD-Analyzer (Faksri et al., 2016). Variant calling for all samples was carried out with ploidy as “1” using GATK HaplotypeCaller (McKenna et al., 2010). Default parameters were used for haplotype calling, viz., base quality score ≥10 and mapping quality ≥20. Independent runs of CombineGVCF followed by genotype calling were carried out for L5 and L6 samples. SNPs pertaining to the PE and PPE regions along with phages and insertion sequences were excluded from the analysis (Stucki et al., 2016). Variants were further filtered to remove SNPs present in only one sample (referred to as “singleton SNPs”), absent in >50% of the samples along with removal of tri- and multi-allelic sites. All SNPs were annotated using SnpEff (version 4.3t) with *Mycobacterium tuberculosis* H37Rv (NC_000962.3) as the reference annotations (Cingolani et al., 2012). SNPs were functionally classified as per their annotations reported in TubercuList (Lew et al., 2011). SNPs that had only a single alternate allele across all samples referred to as “alternate homozygous SNPs” were analyzed (Zojer et al., 2017). Drug-resistant genes were identified from the literature, and SNPs belonging to these genes were annotated (Gygli et al., 2017; Ghosh et al., 2020). The variant calling format (*vcf*) file pertaining to the homozygous SNPs were processed using customized *in-house* generated scripts to obtain FASTA sequences of individual samples. CD-HIT was used for removal of identical sequences (Li and Godzik 2006).

**FIGURE 1:**
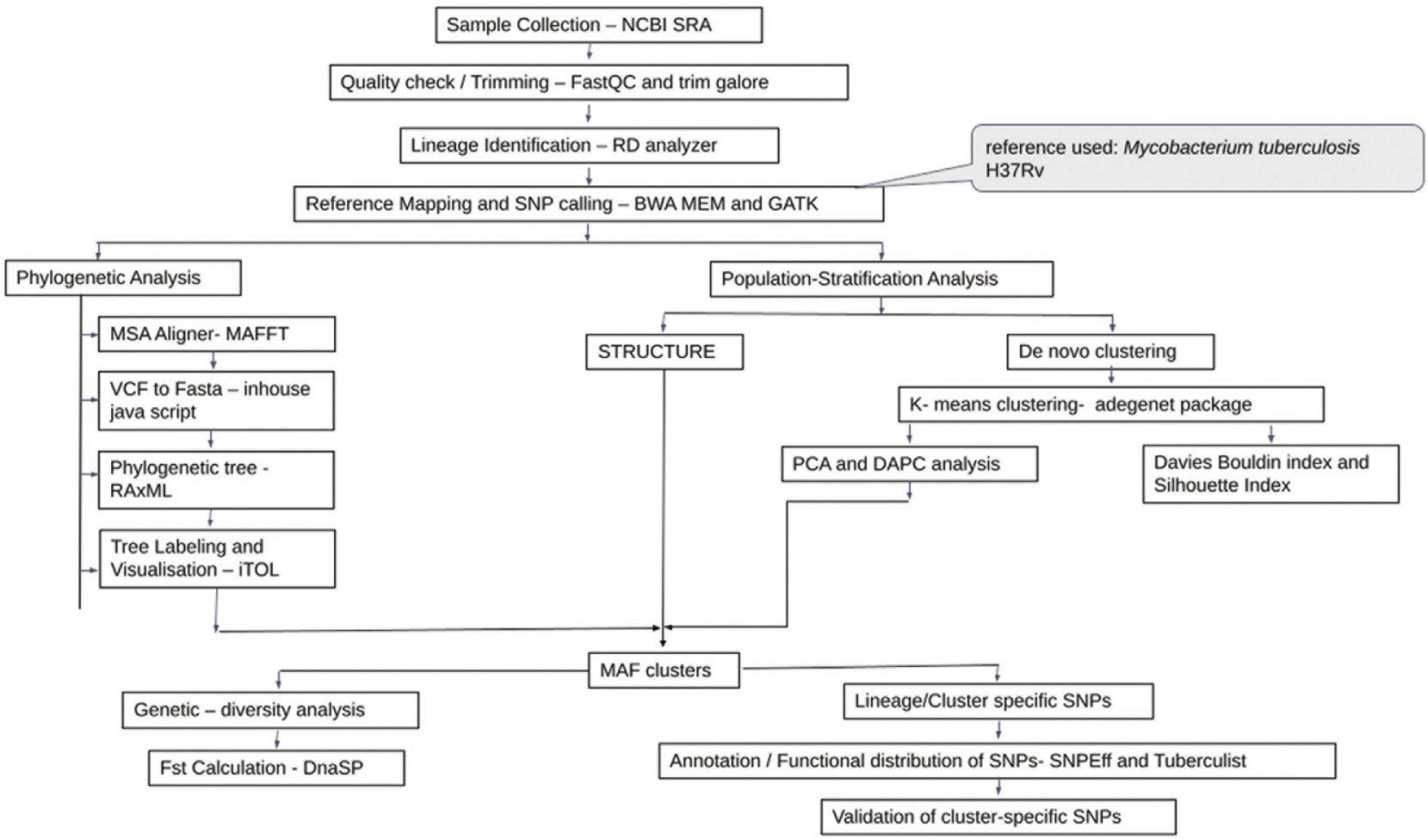
Flowchart for genetic diversity analysis of *Mycobacterium africanum*.

Additional 270 *Maf* samples were used for validation (Supplementary Table S2). A similar protocol for read quality checking and reference mapping was followed as stated previously. Variant calling for the validation set was performed using Pilon version 1.23 (Walker et al., 2014) (Figure 1).

### 2.2 Phylogenetic Analysis

A total of three datasets viz., all *Maf* (L5 and L6) samples (#380 termed as D1), all L6 samples (#197 termed as D2), and all L5 samples (#183 termed as D3) were analyzed independently. Multiple sequence alignment (MSA) of the polymorphic sites of each of these three datasets was obtained using MAFFT (v7.450) (Katoh et al., 2002). Maximum likelihood (ML) phylogenetic tree was generated using parallel MPI implementation of RAxML ver. 8 (Stamatakis, 2014). The general time reversible model of nucleotide substitution under the gamma model of rate heterogeneity (GTRCAT) was used with 1,000 bootstraps (Lanave et al., 1984; Gatto et al., 2007). The trees were visualized using iTOL (Letunic and Bork, 2019).

### 2.3 Population Stratification

Both model and non-model based methods were used for analyzing the underlying population structure of *Maf*. For model-based analysis, ParallelStructure (Besnier and Glover, 2013), which is an implementation of STRUCTURE tool (Pritchard et al., 2000) capable of taking advantage of multi-core computing architecture, was used. R version 3.4.4 and package *parallel_structure* available on a 2 TB RAM Ubuntu 18.04.5 LTS server were used for running the parallelSTRUCTURE tool. Parsimonious informative (PI) sites were derived from multiple genome alignment using MEGAX (Kumar et al., 2018). Linkage equilibrium was estimated using LIAN (Haubold and Hudson 2000) with 10,000 replicates. Admixture and linkage models with correlated allele frequencies were used for population structure estimation. A total of ten independent simulations of Markov chain Monte Carlo (MCMC) were used to derive the optimal number of clusters (*k*) with three sets of burn-in and burn-length (combination of 100,000–300,000; 150,000–350,000; 200,000–400,000). Optimal *k* was chosen based on the Evanno method (Evanno et al., 2005) as implemented in Structure Harvester (Earl and vonHoldt 2012). A cutoff of ≥0.05 was used for membership assignment to a given cluster.

### 2.4 *De Novo* Clustering Methods

Along with model-based approaches such as STRUCTURE and phylogenetic reconstruction, *de novo* method, namely, *K-means* clustering along with other non-model-based approaches requiring *prior* information, viz., discriminant analysis of principal components (DAPC), was also performed on the SNP profile of *Maf* samples. (Jombart et al., 2010; Grünwald and Goss, 2011, Montano et al., 2015). These methods were implemented on SNP data with reduced dimensions using principal component analysis (PCA).

*K-means* clustering was performed using PCA components explaining 95% of the variance in the data and was analyzed on three datasets (D1–D3) independently. To arrive at an optimal number of clusters for each data set, two layers of optimization were applied. First, an elbow plot was obtained, which gave the range of the optimal number of clusters near the elbow. Further on, classification performance measures, such as *Davies Bouldin* and *Silhouette* indices, were calculated. The lowest value of the *Davies Bouldin* index and highest value of the *Silhouette* index estimate the optimal number of *K-means* clustering, as both the indices complement each other.

### 2.5 Population Stratification Using Discriminant Analysis of Principal Components

As an exploratory option, a multivariate method, DAPC available in R package *adegnet,* was also used to infer the genetic structure of the *Maf* datasets (Jombart, 2008; Jombart et al., 2010; Grünwald and Goss, 2011, Montano et al., 2015). The discriminant analysis (DA) method uses populations defined *a priori* to maximize the genetic variation present between groups and minimize the within-group variation (Jombart, 2008). The dimensionality of the data is reduced using PCA followed by assessment of different predefined groups or clusters performed on the basis of DA components, resulting in posterior membership probability value for each sample to a defined cluster. These membership probability values are further analyzed to arrive at the optimal number of clusters where the variation of underlying data can be efficiently explained. The vcf file was read into R by using the *vcfR* tool to create a *vcfR* object (Knaus and Grünwald, 2017). This object was further converted into a *genlight* object using the vcfR2genlight function, providing ploidy information as “1” along with the predetermined population information obtained using STRUCTURE output. The *genlight* object retains only the “alternate homozygous SNPs” in the dataset. PCA was performed using the glPCA function in R. The maximum number of PCA components which explains 95% of the cumulative variance of SNP profiles were taken into account for DA. To avoid overfitting of the data, the optimal number of PCs required to explain the separation of individuals into predefined groups was achieved using the *xval* cross-validation function in R. DAPC was computed using the optimal number of PCs obtained through *xval* cross-validation. To obtain the number of discriminant functions to be retained, F-statistics for DA eigenvalues was calculated. These retained DA components define the membership probability of each sample in the population. The scatter plots and membership probability plots were obtained using the *ggplot2* package in R (Wickham 2016).

### 2.6 Estimation of Genetic Diversity for the Clusters Obtained

Fixation index (*Fst*) and average pairwise nucleotide diversity indices per site ( π ) was calculated in order to measure the robustness of the clusters obtained with parameters set as “haploid” and “prokaryotes” using DnaSP ver. 6.12.03 (Rozas et al., 2017).

### 2.7 Identification of Cluster-Specific Unique Single Nucleotide Polymorphisms

**FIGURE 2:**
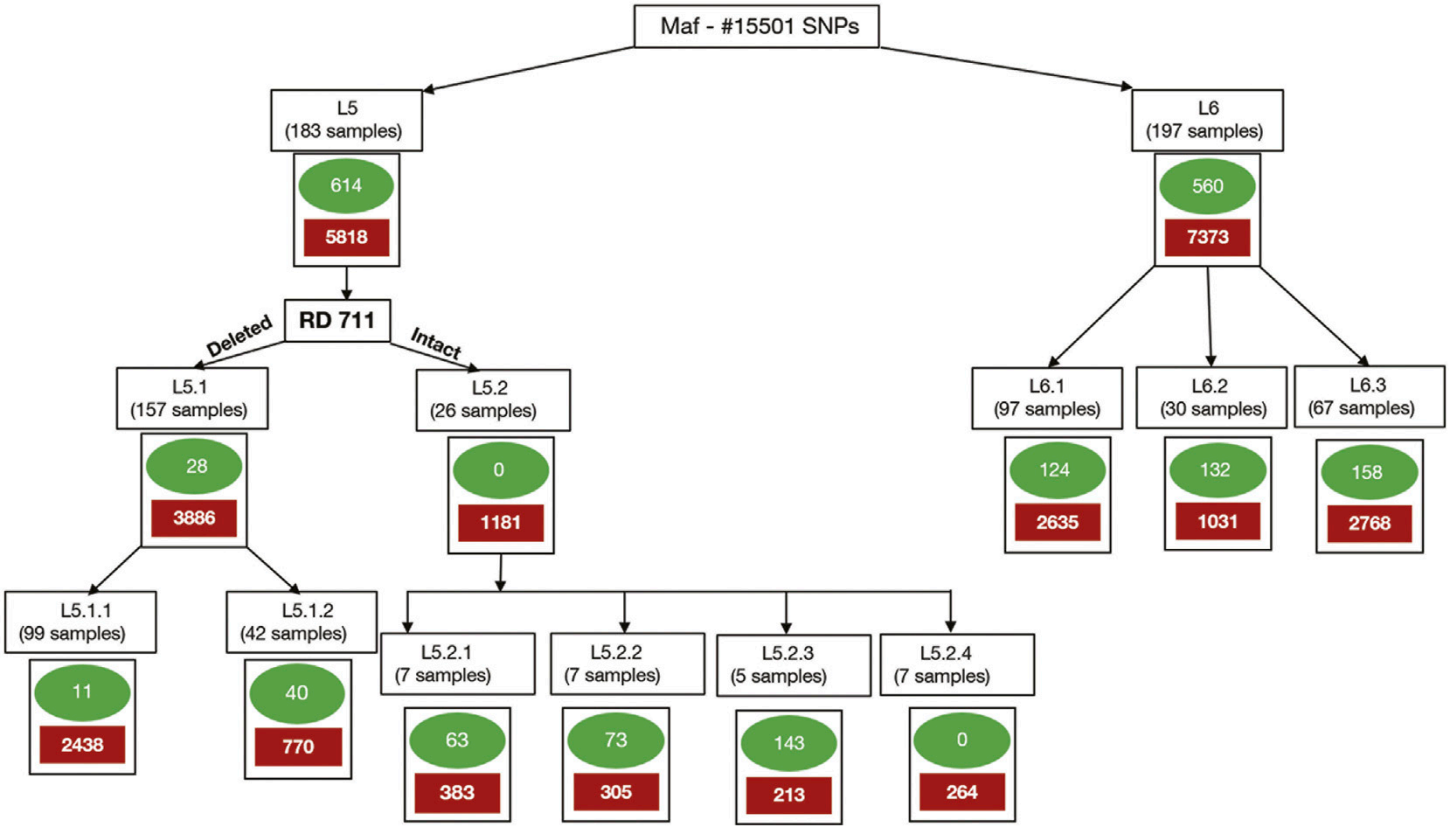
Methodology for identification of core-cluster-specific SNPs. *Cluster-specific-core* SNPs are represented by green ovals, and *cluster-specific-total* SNPs are represented by solid red boxes.

Clusters/sub-populations in the given dataset were identified using a combination of phylogenetic and population stratification analysis. SNPs present in at least one isolate of a cluster and absent in members across other clusters are termed “*total-cluster-specific*” SNPs. *“Core-cluster-specific*” SNPs were derived using “*total-cluster-specific*” SNPs with additional criteria of the SNPs being present across all samples in the cluster. The “*core-subcluster-specific-SNP*” was derived from the “*total-subcluster-specific-SNPs*” using the same criteria of SNP being present in all members of the sub-cluster (Figure 2). Functional annotations were carried out using SnpEff (Cingolani et al., 2012), Tuberculist (Lew et al., 2011), Kyoto Encyclopedia of Genes and Genomes (Kanehisa et al., 2017), and BioCyc (Karp et al., 2019). *Core-cluster-specific* SNPs were also analyzed in the context of previous studies (Ates et al., 2018; Coscolla et al., 2021). Synonymous SNPs obtained for each cluster were used as biomarkers for the identification of *Maf* samples.

### 2.8 Validation of Cluster-Specific Single Nucleotide Polymorphisms

*Core-cluster-specific* synonymous SNPs were used to validate the (sub)-lineage identity in a validation dataset of 270 samples. Synonymous SNPs were preferred because these are under relatively lower selection pressure (Coll et al., 2014). Synonymous SNPs associated with drug-resistant genes were demarcated and validation was carried out, both including and excluding these SNPs. An additional criterion of occurrence of RD711 was used for validating L5 samples. The SNPs obtained for L6 clusters were also mapped with existing growth attenuation and expression studies (Gehre et al., 2013; Ofori-Anyinam et al., 2017).

## 3 Results

### 3.1 Reference Mapping, Filtering and Variant Calling

Of the 572 *Maf* samples, 497 passed the quality check and 75 needed trimming because of poor quality. Of the 75, 18 were discarded due to insufficient read lengths after trimming. The remaining 554 were used for lineage identification based on RD regions. Of these, 235 samples belonged to L6 lineage, and 173 samples were identified as belonging to L5 lineage. The remaining 34 samples could not be identified based on RD-regions and were termed “unidentified” (Supplementary Table S1). Further removal of samples that did not fit the DP and MQ criteria resulted in a total of 157 (L5), which also harbored the RD711 deletion and 192 (L6) samples and were used for SNP identification. Of the unidentified samples, three were not included in the present study (termed “*intermediates*”) as they were found to branch independently between L5 and L6 samples in the phylogenetic tree (Supplementary Table S1 and Supplementary Figure S1). All samples, including 31 unidentified, were further subjected to variant calling, which revealed a total of 38,343 variants. Variants were filtered according to the criteria mentioned in the Methods section, which resulted in a total of 15,501 SNPs that were used for downstream analysis.

### 3.2 Phylogenetic Analysis

**FIGURE 3:**
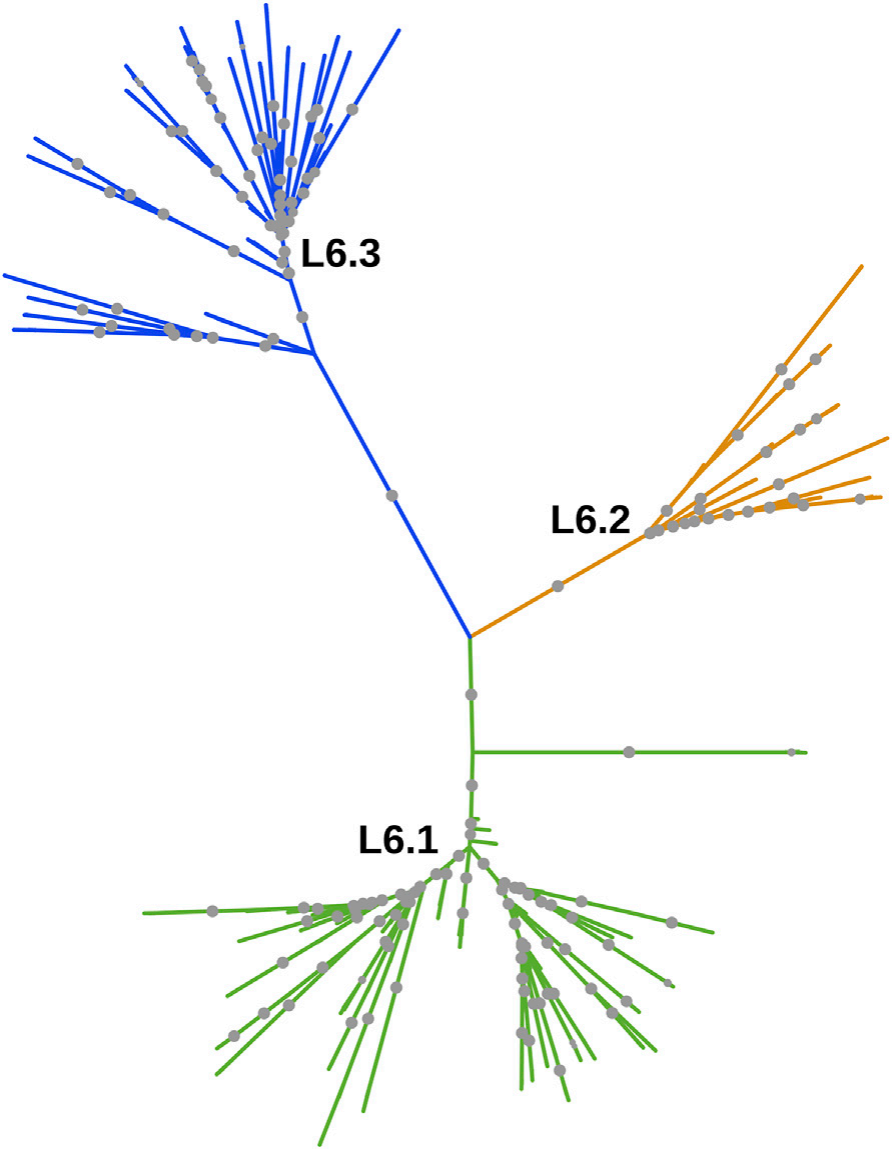
Phylogenetic tree of *Mycobacterium africanum* L6 samples derived using the maximum likelihood method, as implemented in RAxML based on genome-wide SNPs. Significant bootstrap values > 70% are represented by solid gray circles.

**FIGURE 4:**
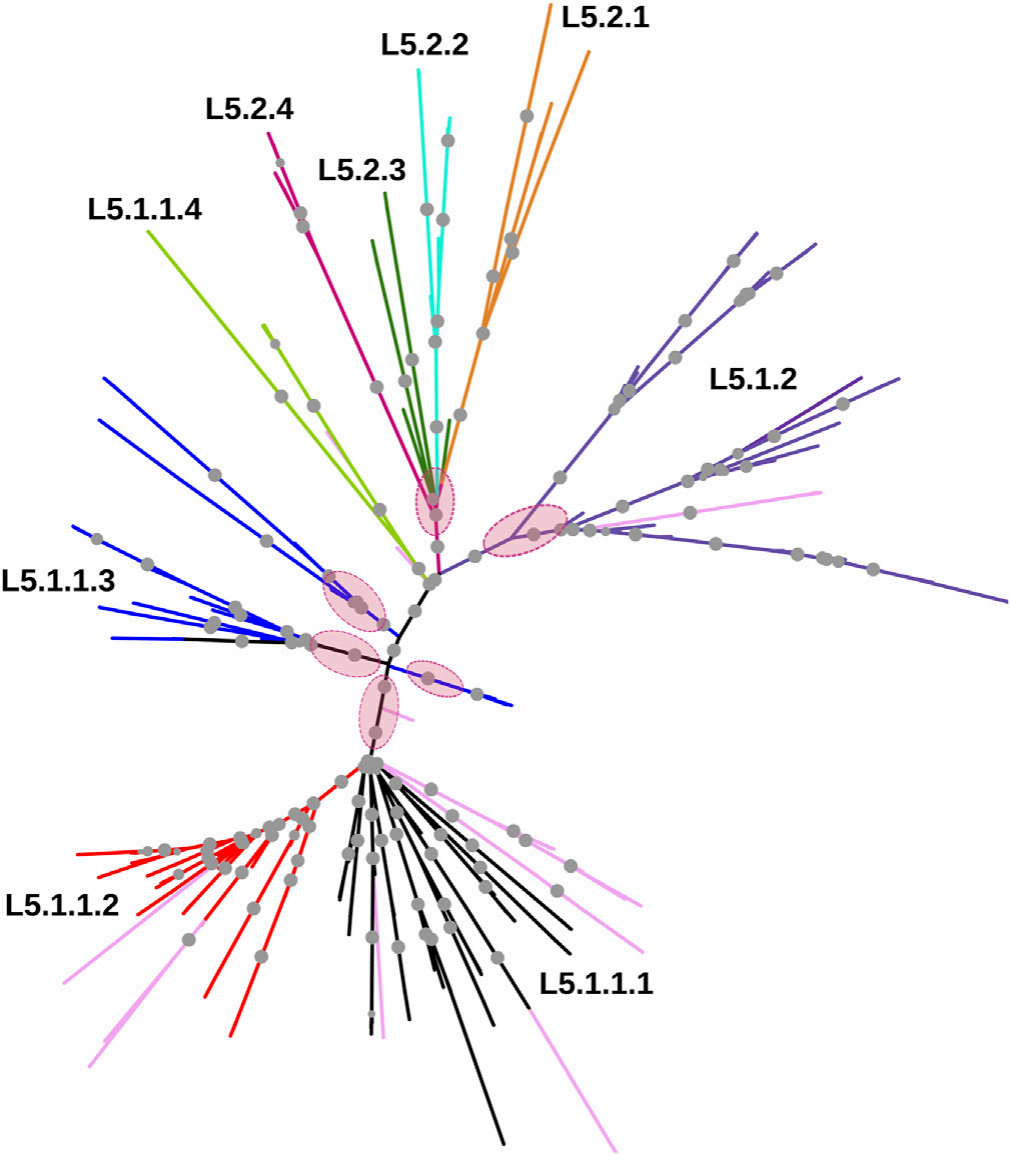
Phylogenetic tree of *Mycobacterium africanum* L5 samples derived using the maximum likelihood method, as implemented in RAxML based on genome-wide SNPs. Pink circles represent the six major monophyletic clusters observed. Samples marked in light pink represent admixed samples as reported by the STRUCTURE tool. Significant bootstrap values >70% are represented by solid gray circles.

Whole-genome phylogeny revealed lineage-wise (L5 and L6) clustering of samples (Supplementary Datasheet S1). In the L6 cluster (in particular L6.3), five unidentified samples lacked RD702 (SRA Accession ID: ERR751293, SRR998600, SRR998602, SRR998741, and SRR998742). Furthermore, three samples were found to branch out independently, serving as intermediaries between the L5 and L6 clusters, and as explained earlier, were excluded for further analysis (Supplementary Figure S1). The rest of the unidentified 26 samples, which lacked the RD711 region, were found to cluster along with the L5 samples. Phylogenetic analyses of L6 samples revealed three independent monophyletic clusters representing L6.1, L6.2, and L6.3 sub-lineages (Figure 3). In addition, a small monophyletic cluster (SRA Accession ID: SRR1162716, SRR998647, and SRR998646) was found at the base of L6.1. In the case of the L5 phylogenetic tree, six major monophyletic clusters were observed. Seven samples (SRA Accession ID: ERR1023216, ERR1082139, ERR751335, ERR751290, ERR702413, ERR751343, and ERR751322) were found to cluster as the outermost branch of the 26 unidentified samples which lacked RD711 deletion (Figure 4).

### 3.3 Population Stratification Analysis Using STRUCTURE

**FIGURE 5:**
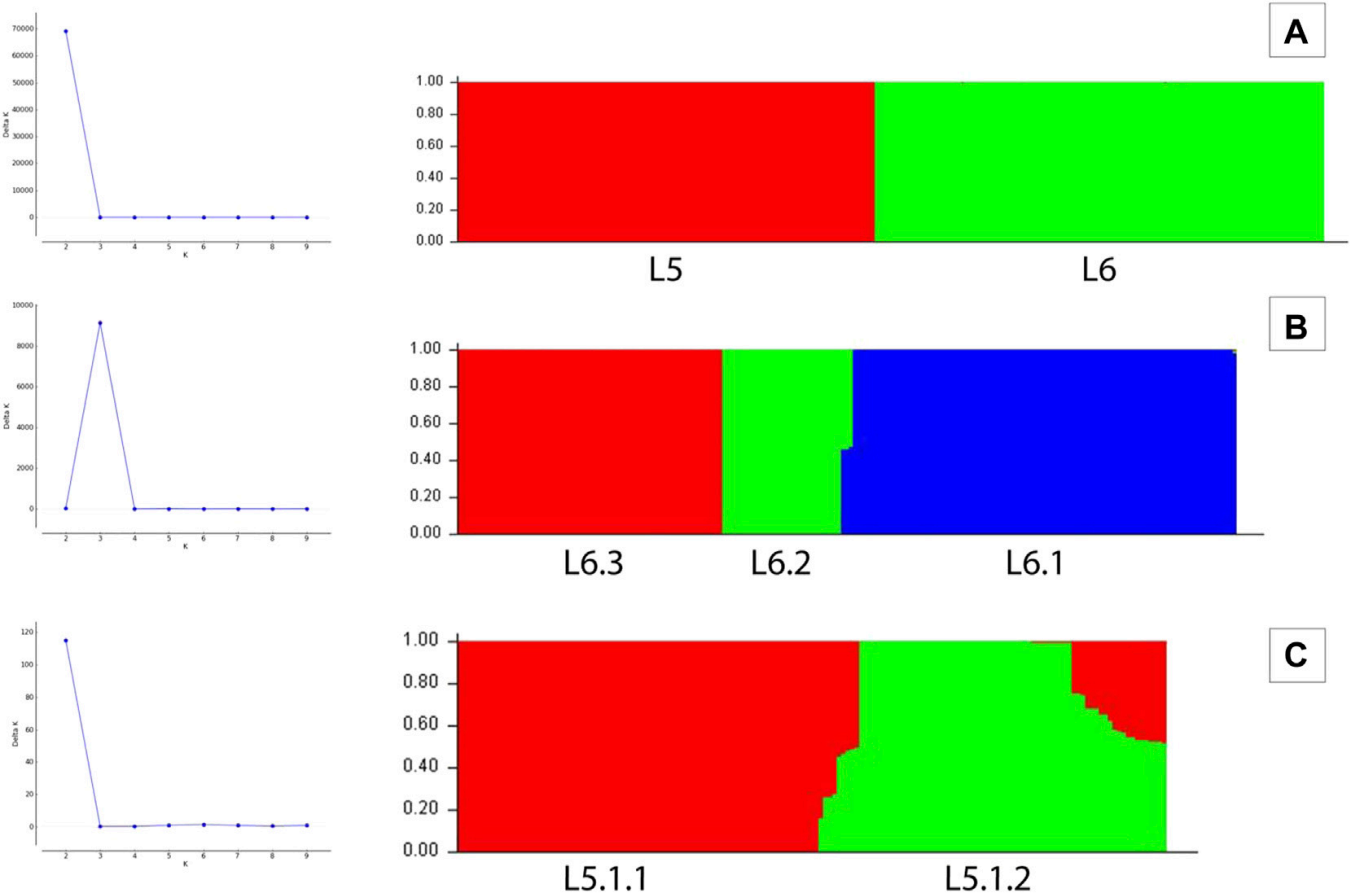
**(A–C)** Population stratification of *Mycobacterium africanum* based on genome-wide SNPs [**(A)** population structure of 380 samples of lineages L5 and L6 at *k* = 2; **(B)** population structure of 197 samples of lineage L6 at *k* = 3; **(C)** population structure of 157 samples of sub-lineage L5.1 at *k* = 2].

A set of 15,390 PI sites belonging to 380 *Maf* samples (dataset D1) were obtained from the multiple genome alignment (Table 1). Linkage disequilibrium calculated in terms of I^S^_A_ was found to be 0.15. Population structure analysis revealed an optimal peak at *k* = 2 which corresponds to L5 and L6 lineages, respectively (Figure 5A, Supplementary Table S3). A set of 7,373 SNPs for L6 (dataset D2) and 5,818 SNPs for L5 (dataset D3), respectively, was used for the identification of PI sites independently (Table 1). Fine-level clustering of L6 lineage (dataset D2 with 7028 PI sites) revealed an optimal peak at *k* = 3 that corresponds to three independent sub-lineages viz., L6.1 (100 samples), L6.2 (30 samples), and L6.3 (67 samples) (Figure 5B) while three samples were found to be admixed with relatively higher membership to L6.1 and lower membership to L6.2 (Supplementary Table S4). L5 samples did not cluster as per the occurrence of RD711 in the absence of prior population information. Hence, RD711 was used as a marker to demarcate L5 lineage (Comín et al., 2021). This resulted in two groups (Group 1 with RD711: 157 samples and Group 2 without RD711: 26 samples), each of which was then subjected to population stratification analysis. A total of 4,633 and 1,931 SNPs for L5.1 and L5.2, respectively, were used for the identification of PI sites independently (Table 1). Group 1 (157 samples, 4147 PI sites) had an optimal peak at *k* = 2 and a minor peak at *k* = 6 (Figure 5C). The membership coefficients at *k* = 2 revealed that the two clusters correspond to L5.1.1 and L5.1.2 while 16 samples were found to be admixed, of which seven (ERR751315, ERR702407, ERR439931, ERR1023217, ERR1082137, ERR751310, and ERR702409) were found to share major membership to L5.1.1 and nine (ERR1023216, ERR751290, ERR751304, ERR751305, ERR702413, ERR751322, ERR751343, ERR1082139, and ERR751335) were found to share major membership to L5.1.2 (Figure 5C, Supplementary Table S5).

**TABLE 1:** Details of datasets and their corresponding total number of SNPs and PI sites used for the study.

Dataset	Lineage/sub-lineage	# Samples	# SNPs	# PI sites
D1	L5 and L6	380	15,501	15,390
D2	L6	197	7,373	7,028
D3	L5	183	5,818	5,436
Group 1	L5.1	157	4,633	4,147
Group 2	L5.2	26	1931	1,292

In case of L5.1, the minor peak at *k* = 6 revealed five clusters based on major membership coefficient values >0.9 with the sixth cluster having membership coefficient ≤0.3 (L5.1.1_minor) (Supplementary Figure S2A, Supplementary Table S6). Of the five clusters, four subdivided L5.1.1 into L5.1.1.1 (#29 samples) with 13 admixed (12 samples having major and minor membership to L5.1.1.1 and L5.1.1_minor cluster, respectively, and one sample having major and minor membership to L5.1.1.1 and L5.1.1.3, respectively), L5.1.1.2 (#36 samples) with three admixed (major and minor membership to L5.1.1.2 and L5.1.1_minor cluster, respectively), L5.1.1.3 (#27 samples) with one admixed (ERR1023216 having major membership to L5.1.1.3 and minor membership to L5.1.2 and L5.1.1.4), and L5.1.1.4 (#5 samples) with one admixed (ERR751290 having major membership coefficient to L5.1.1.4 and minor membership to L5.1.1.3). The six major monophyletic clusters observed in case of the L5 phylogenetic tree corroborated with the five clusters of L5.1 (L5.1.1.1–L5.1.1.4 and L5.1.2) and one cluster of L5.2, of which cluster L5.1.1.4 corresponds to the outermost branch of L5.2 samples (Figure 4). The fifth cluster corresponds to L5.1.2 (#40 samples) with two admixed (ERR439949 and ERR439973 having major and minor membership coefficients to L5.1.2 and L5.1.1.4, respectively) (Supplementary Table S6). Group 2 (26 samples, 1292 PI sites) had an optimal peak at *k* = 4 which corresponds to L5.2.1–L5.2.4 sub-lineages with one admixed sample (Supplementary Figure S2B, Supplementary Table S7).

### 3.4 Sub-Lineage Mapping With Previous Studies

The observed L6 sub-lineages agreed with previous study (Coscolla et al., 2021). L5 had been classified into three groups, namely, L5.1, L5.2, and L5.3 by Coscolla et al. (2021) (Supplementary Table S8). The further classification of the L5.1 sub-lineage proposed in this study is in partial agreement with that reported by Coscolla et al. (2021) (Table 2, Supplementary Table S8). L5.2 and L5.3 of Coscolla et al. (2021) correspond to L5.2.1 and L5.2.2 (proposed in this study). It is worth noting that members belonging to L5.2.3 and L5.2.4 (proposed in this study) remained unassigned in earlier studies (Coscolla et al., 2021) (Supplementary Table S9). L5.2 of Ates et al. (2018) also corresponds to L5.2.1 proposed in this study.

**TABLE 2:** Comparison of L5 (sub)-lineage mapping with previous studies.

Lineage reported in previous studies	Lineage identified in current study using STRUCTURE (*k* = 2)	Lineage identified in current study using STRUCTURE (*k* = 6)
L5.1 (Group 1) samples with RD711		
L5.1.1	L5.1.1	L5.1.1.1/L5.1.1.2
L5.1.2	L5.1.1	L5.1.1.3
L5.1.3	L5.1.1	L5.1.1.3
L5.1.4	L5.1.2	L5.1.2
L5.1.5	L5.1.2	L5.1.2
NA	L5.1.1	L5.1.1.4
**Lineage reported in previous studies**	**Lineage identified in the current study using STRUCTURE (*k* = 4)**	
L5.2 (Group 2) samples without RD711		
L5.2	L5.2.1	
L5.3	L5.2.1	
NA	L5.2.3 and L5.2.4	

### 3.5 Population Stratification Analysis Using *De Novo* Clustering Methods

**FIGURE 6:**
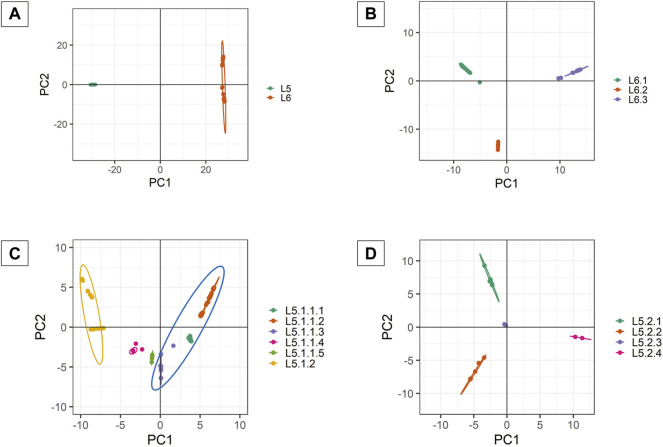
**(A–D)** PCA plots of *Mycobacterium africanum* derived using genome-wide SNPs [**(A)** PCA distribution of 380 samples belonging to lineages L5 and L6; **(B)** PCA distribution of 197 samples belonging to lineage L6; **(C)** PCA distribution of 157 samples belonging to sub-lineage L5.1; **(D)** PCA distribution of 26 samples belonging to sub-lineage L5.2].

PCA analysis of dataset D1 (L5 and L6) (Table 1) revealed a total of 38 PCs accounting for 95% of the total variability in the data which was selected for further analysis (Supplementary Figure S3). The first PC (∼80% variance) differentiated D1 into L5 and L6 lineages (Figure 6A). This agrees with the optimal value of *k* = 2 obtained using *K-means* clustering optimization methods (Supplementary Table S10A–C). Further PCA analysis of dataset D2 (L6) revealed three distinct sub-clusters, namely, L6.1, L6.2, and L6.3 (Figure 6B), that find agreement with *K-means* (Supplementary Figure S4, Supplementary Table S11A–C). PCA and *K-means* clustering approaches of the dataset D3 (L5) remained inconclusive. Hence, clustering of L5 samples using prior knowledge of the occurrence of RD711 was carried out (Comín et al., 2021). Group 1 consisting of 157 L5 samples with the presence of RD711 marker, revealed two major clusters differentiated on PC1 (∼24% variance) (Figure 6C). Taking into account the variation along PC2 (∼8% variance) six sub-clusters were obtained which partially supported the minor peak (*k* = 6) obtained using STRUCTURE (Figure 6C, Supplementary Figure S5). Of the six sub-clusters, five corroborated with earlier reports (Coscolla et al., 2021), with an additional sub-cluster, namely L5.1.1.4, reported exclusively in this study (Figure 6C, Supplementary Tables S6, S8). Group 2 consisting of 26 L5 samples with the absence of RD711 marker, revealed the presence of four distinct clusters across PC1 (∼24% variance) and PC2 (∼22% variance) (Figure 6D, Supplementary Figure S6, Supplementary Tables S7, S9). However, *K-means* clustering could not resolve the L5 sub-lineages based on RD711.

### 3.6 Population Stratification Analysis Using Discriminant Analysis of Principal Components

DAPC clustering for all the datasets (D1, D2, and D3) (Table 1) was performed on *a priori* clustering information obtained from phylogeny, STRUCTURE, and PCA. Therefore, *k* = 2 was chosen for DAPC analysis of dataset D1. Along PC1, the *Maf* samples are differentiated into two clusters. Intra-population differentiation for L6 was observed along the PC2 axis (Figure 6A). Based on *xval* cross-validation, 5 PCs (88.7% of the total variance) and one discriminant eigenvalue were found to explain the two distinct clusters obtained, viz., L5 and L6 (Supplementary Figures S3, S7, S8). The lineage-wise clusters obtained were further used for fine-grain clustering to extract the sub-lineages present, if any.

DAPC clustering of the dataset D2 (L6) was carried out at *k = 3* using 54 PCs (∼95% variance), and the resulting clusters are in agreement with that obtained from other approaches (Figure 6B, Supplementary Figure S9). Cross-validation determined 10 PCs (∼69.44% variance) to be optimal along with two discriminant eigenvalues that explain three distinct sub-clusters of L6, namely L6.1, L6.2, and L6.3 (Supplementary Figures S9, S10).

Group 1 and 2 of L5 samples, viz., L5.1 (with RD711) and L5.2 (without RD711), were subjected to DAPC clustering independently using *k = 2* and *k = 4,* respectively. A total of 55 PCs (∼95% variance) were used for clustering L5.1, whereas 12 PCs (95% of the variance) were used for Group 2 (Figure 6C, Supplementary Figure S5). Cross-validation indicated 50 PCs (∼94% variance) and 4 PCs (64.94% of the total variance) to be optimal for Group1 and Group2, respectively (Supplementary Figures S11, S12). DAPC analysis using 50 PCs and one discriminant eigenvalue was found to explain two distinct sub-clusters obtained for Group1, namely L5.1.1 and L5.1.2 (Supplementary Figure S13). Similarly, four optimal PCs and two discriminant eigenvalues explained four distinct sub-clusters for Group2, namely L5.2.1, L5.2.2, L5.2.3, and L5.2.4 (Supplementary Figure S14).

### 3.7 Estimation of *Maf* Genetic Diversity

The highest genetic differentiation was observed between L5 and L6 lineages (*Fst* = 0.89). The population differentiation index for the three L6 sub-lineages varied from 0.59–0.65 (Supplementary Table S12). *Fst* between L5.1.1 and L5.1.2 was 0.46, whereas, for the four L5.2 sub-clusters it was found ranging from 0.39–0.74. In the case of five L5.1 sub-clusters *Fst* from 0.3–0.64 (Supplementary Table S12).

The whole-genome average pairwise nucleotide diversity (π) within the L6 samples was found to be 0.06, whereas the same within L5 samples was found to be 0.05, which supports the fact that L6 is more genetically diverse than L5. Furthermore, π was reported to vary between 0.076–0.18 for the L6 sub-clusters (Supplementary Table S13). π within the L5.1 and L5.2 clusters were found to be 0.06 and 0.04, respectively. Within cluster variation between L5.1 and L5.2 sub-clusters were also studied, which revealed the highest nucleotide diversity of 0.48 for the L5.2.4 sub-cluster. Based on the sub-optimal peak for L5.1 (*k* = 6) obtained in STRUCTURE, π was calculated for each of the sub-clusters. With the removal of two admixed samples from L5.1.2, the π reduced from 0.23 to 0.13 (Supplementary Table S13).

### 3.8 Identification of Core-Cluster-Specific Single Nucleotide Polymorphisms

Total-cluster-specific SNPs identified in L5 are 5,818 which belong to 2,553 genes. Similarly, for L6, 7,373 SNPs belonging to 2,835 genes were identified (Table 1). Genes involved in drug resistance are also part of this set. Drug-resistant TB is a major challenge, and hence genes involved in drug resistance in *Maf “total-cluster-specific-SNPs”* were annotated even though drug resistance is rare in *Maf* as compared to *M.tuberculosis* (Asante-Poku et al., 2015; Acquah et al., 2021). A total of 24 genes involved in drug resistance that contain 79 SNPs were found to be part of L6-“*total-cluster-specific*” SNPs (Supplementary Table S14). Similarly, in case of L5, 20 drug-resistant genes containing 69 SNPs were found to be part of L5- “*total-cluster-specific*” SNPs (Supplementary Table S15).

#### 3.8.1 L6-Specific Single Nucleotide Polymorphisms

A total of 602 L6-specific SNPs were identified, of which 331 are missense and seven are stop-gained while the rest are synonymous and upstream SNPs (Figure 2; Table 3, Supplementary Table S14). Population stratification analysis of L6 samples revealed three sub-lineages L6.1, L6.2, and L6.3 for which sub-cluster specific SNPs 123, 132, and 158 were identified (Table 3, Supplementary Table S14). The identified sub-cluster specific SNPs (obtained in our study) mapped onto *Maf* samples of respective sub-lineage as detailed by Coscolla et al. (2021). Coscolla et al. (2021) also reported a set of sub-lineage specific SNPs, which remained unmapped with the “*core-cluster-specific*” SNPs obtained in our study (Supplementary Figure S15) but mapped with the “*total-cluster-specific*” SNPs for the L6 dataset. This highlights the fact that the sub-lineage specific SNPs of Coscolla et al., 2021 do not satisfy the criteria of being present across all samples. The synonymous SNPs identified in our study were used for the validation of sub-lineage specificity (Supplementary Table S14).

**TABLE 3:** Summary of L5 and L6 (sub)cluster-specific-core SNPs.

Functional annotation	L5	L5.1.1	L5.1.2	L5.2.1	L5.2.2	L5.2.4	L6	L6.1	L6.2	L6.3
Synonymous										
Cell_wall	453	1	4	12	8	14	573	8	6	10
Conserved_hypothetical	382	2	6	4	5	13	560	8	13	13
Lipid_metabolism	255	0	1	1	5	4	289	4	4	5
Pathways	146	0	1	2	0	2	192	10	11	15
Regulatory_proteins	95	0	1	2	1	3	108	4	4	2
Metabolism_respiration	577	2	2	4	9	23	699	2	1	2
Virulence	89	0	2	0	1	2	109	2	3	1
Missense										
Cell_wall	686	1	6	7	3	16	877	11	17	25
Conserved_hypothetical	686	2	2	9	8	16	885	15	12	20
Lipid_metabolism	311	0	3	2	4	4	419	7	7	7
Pathways	193	0	1	3	4	3	271	23	21	17
Regulatory_proteins	150	0	2	0	1	5	207	6	6	9
Metabolism_respiration	804	2	3	11	10	17	1,017	5	6	6
Virulence	145	0	2	1	2	3	175	3	7	3
Upstream/downstream										
Cell_wall	137	0	0	2	3	6	191	2	3	8
Conserved_hypothetical	217	0	0	2	2	2	218	5	5	3
Lipid_metabolism	52	0	0	0	0	0	69	0	0	3
Pathways	47	0	0	0	1	0	52	3	2	5
Regulatory_proteins	37	0	0	0	0	0	54	1	2	1
Metabolism_respiration	137	1	3	0	3	6	193	1	1	1
Virulence	26	0	0	0	1	1	30	1	0	0
Stop gained/lost/spliced										
Cell_wall	25	0	0	0	1	0	37	0	0	1
Conserved_hypothetical	40	0	1	1	0	1	55	1	0	0
Lipid_metabolism	7	0	0	0	0	1	17	0	0	0
Pathways	2	0	0	0	0	0	4	0	0	0
Regulatory_proteins	8	0	0	0	0	0	9	0	0	0
Metabolism_respiration	25	0	0	0	1	1	34	1	1	1
Virulence	5	0	0	0	0	0	8	0	0	0
Total	5,737 + 81(transcript _variants)	11	40	63s	73	143	7,352 + 21(transcript_variants)	123 + 1(transcript_variant)	132	158

Functional mapping of “*core-cluster-specific*” missense SNPs of L6 and its sub-lineages was carried out to understand its role in growth attenuation and adaptation to hypoxia (Gehre et al., 2013; Ofori-Anyinam et al., 2017; Ofori-Anyinam et al., 2020) (Table 4, Supplementary Table S16). It is interesting to note that all genes harboring the missense mutations were found to have lower expression in L6, which aid growth in microaerophilic environments (Ofori-Anyinam et al., 2017).

**TABLE 4:** Functional mapping of core-cluster-specific missense SNPs of L6 with literature support (Gehre et al., 2013; Ofori-Anyinam et al., 2017; Ofori-Anyinam et al., 2020) functional role obtained from Mycobrowser (url: https://mycobrowser.epfl.ch/).

Lineage/sub-lineage	Rv locus/gene name	Functional role	SNP
L6	Rv0862c	Conserved hypothetical protein	Asp160Glu
	Rv1096	Probably involved in carbohydrate degradation	Pro272Ser
	Rv2241/aceE	Involved in energy metabolism	Ala777Thr
	Rv2383c/mbtB	Involved in biogenesis of siderophore mycobactins	Leu978Phe
	Rv2737c/recA	Involved in regulation of nucleotide excision repair	Gln566Pro
	Rv2194/ *qcrC*	Required during aerobic respiration for growth; may be responsible for differential energy metabolism	Lys228Gln
	Rv1023/eno	Role in tissue re-modeling and invasion of host cells; a potential drug target (Rahi et al., 2017)	Arg179Ser
	Rv1240/mdh	Involved in tricarboxylic acid cycle	Asp253Ala
	Rv0066c/icd2	Involved in tricarboxylic acid cycle	Lys117Asn
L6.1	Rv3563/fadE32	Involved in lipid degradation	Glu206Val
	Rv0080	Conserved hypothetical protein	Val31Gly
	Rv2504c/scoA	Involved in fatty acid degradation/synthesis	Arg230Trp
	Rv3223c/sigH	Alternative sigma factor that plays a role in oxidative-stress response	Glu151Asp
	Rv0066c/icd2	Involved in tricarboxylic acid cycle	Lys117Asn
			Val447Met
	Rv1328/glgP	Phosphorylase is an important allosteric enzyme in carbohydrate metabolism	Gly731Asp
	Rv2112c/dop	Deamidase of prokaryotic ubiquitin-like-protein	Ala500Val
	Rv3282	Conserved hypothetical protein	Thr145Lys
	Rv1178	Probably involved in cellular metabolism	Arg247Arg^a^
	Rv3236c/kefB	Growth attenuation	Arg325His
L6.2	Rv3236c/kefB	Growth attenuation	Val106Ala
	Rv2215	Involved in tricarboxylic acid cycle and antioxidant defense	Ala338Val
	Rv1121/zwf1	Involved in the pentose phosphate pathway	Gln277*
L6.3	Rv1180/pks3	Potentially involved in intermediate steps for the synthesis of polyketide	Pro401Thr
	Rv1181/pks4	Involved in lipid metabolism	Gly40Arg
	Rv 2030c	Conserved hypothetical protein	Ser275Asn
	Rv1447c/zwf2	Involved in pentose phosphate pathway	Gly357Ser

a Absent in only one isolate (SRA Accession ID: SRR1577833).

*Stop codon

#### 3.8.2 L5-Specific Single Nucleotide Polymorphisms

A total of 648 SNPs were found to be unique to the L5 cluster. Of these, 331, 7, 191, and 7 were found to be missense, stop-gained, synonymous, and upstream SNPs, respectively. A total of 11 *core-cluster-specific* SNPs were identified for the L5.1 sub-cluster; however, none were found for the L5.2 sub-cluster (Figure 2; Table 2, Supplementary Table S15).

It should be noted that 57 and 68 L5.2-specific SNPs reported by Coscolla et al. (2021) and Ates et al. (2018), respectively, are in agreement with our findings of L5.2.1 *core-cluster-specific-SNPs* (#63), except 9 SNPs belonging to PE-PPE/repeat region of Ates et al. (2018). Additional six SNPs, identified in the current study, viz., Ala126Ala (Rv1558), Trp687* (Rv 2082), Ala120Val (Rv2205c), upstream SNP −4,469 (Rv3424c), Gly236Asp (Rv3710), and Gly81Gly (Rv3792) are also found to be present across all L5.2 samples (as per nomenclature given by Coscolla et al. (2021) and isolates characterized in their study) (Supplementary Figure S16, Supplementary Table S15).

Similarly, previously identified L5.3-specific SNPs [#62 as reported by Coscolla et al. (2021)] are also in agreement with our findings of *core-L5.2.2-specific* SNPs (#73) with an exception of Ala189Glu (Rv0180c). Additional 12 SNPs [Tyr126* (Rv0217c), Val174Leu (Rv0809), Ala69Glu (Rv0848), Phe249Phe (Rv0930), Arg302Arg (Rv1188), and Cys34Phe (Rv1317c), upstream SNP -3721 (Rv1749c), Asn372Ser (Rv2874), Gly18Asp (Rv3251c), Asn234Asn (Rv3534c), Ser84Gly (Rv3608c), and Ile150Thr (Rv3842c)] were reported in our study which were found to be absent in L5.3 (#19 of the total 25 L5.3 isolates reported by Coscolla et al. (2021)) (Supplementary Figure S16, Supplementary Table S15). It is worth noting that no unique SNPs were found to be present in the L5.2.4 cluster.

The synonymous SNPs for L5 (sub)lineages were used for the validation of sub-lineage specificity (Supplementary Table S15). Earlier reported L5 (#12 SNPs) and L6 (#10 SNPs) specific synonymous biomarker SNPs were found to be in complete agreement with our study (Napier et al., 2020). The *Maf* (sub)lineage-specific SNPs reported in the current study were found to be exclusive and did not show any match with the previously reported MTBC-lineage specific SNPs listed by Napier et al. (2020).

Functional mapping of L5-“*core-cluster-specific*” missense SNPs was carried out to understand genotype-phenotype correlation (Ofori-Anyinam et al., 2020) (Table 5, Supplementary Table S15).

**TABLE 5:** Functional mapping of core-cluster-specific missense SNPs of L5 with literature support (Ofori-Anyinam et al., 2020), and functional role obtained from MycoBrowser (url: https://mycobrowser.epfl.ch/).

Rv locus/gene name	Functional role	SNP	Additional functional evidence
Rv0211/ pckA	Gluconeogenesis; virulence and initiation of infection in macrophages	Lys422Thr	Collins et al. (2002)
			Liu et al. (2003)
Rv2967c/pca	Gluconeogenesis; cholesterol detoxification and lipogenesis during intracellular growth	Ala926Thr	—
Rv1188/pruB	Proline metabolism associated with attenuated growth and adaptation to hypoxia	Arg257Cys	Griffin et al. (2011)
			Zhang et al. (2012)
			Berney and Cook (2010)
			Giffin et al. (2016)
Rv1552/frdA	Associated with hypoxia and microaerophilic adaptation	Gly16Asp	—
Rv1309/atpG	Produces ATP from ADP in the electron transport chain	Tyr220Ser	—
Rv1307/atpH	Produces ATP from ADP in the presence of a proton or sodium gradient	Ser434Leu	—
Rv1240/mdh	Catalyzes the reversible oxidation of malate to oxaloacetate	Leu326Ile	—
Rv0066c/icd2	Catalyzes the conversion of isocitrate to ɑ-ketoglutarate	Ala455Val	—
Rv0946c/pgi	Central carbohydrate metabolism	His6Tyr	—
Rv1131/prpC	Involved in the methyl citrate cycle	Glu255Ala	—
Rv2454c	Central carbohydrate metabolism	Ala309Gly	—
Rv1127c/Ppdk	Catalyzes the reversible phosphorylation of pyruvate and phosphate	Val425Ala and Ser112Ala	—
Rv3293/Pcd	Involved in L-alpha-aminoadipic acid biosynthesis	Gly198Ala	—

#### 3.8.3 Validation of Cluster-Specific Single Nucleotide Polymorphisms

Variant calling was performed for the validation dataset of 270 samples and filtered using MQ and DP. Every sample in the validation dataset was then classified based on the presence of “*sub-cluster specific synonymous SNP set*” which helped in lineage/sub-lineage assignment. Validation was carried out by both including and excluding the SNPs associated with genes involved in drug resistance and the results were found to be consistent. Of the 270 samples in the validation dataset, 85 and 185 were identified as L5 and L6, respectively. Of the 85 L5 samples, 68 and 13 were identified as L5.1.1 and L5.1.2, respectively. These samples were also found to harbor the RD711 deletion as re-verified through RD-based studies. Of the remaining four samples, one and three were classified as L5.2.1 and L5.2.2, respectively. All the L5.2 samples lacked RD711 deletion, as confirmed by RD-based studies. Of the 185 L6 samples, 131, 20, and 34 were identified as L6.1, L6.2, and L6.3, respectively (Supplementary Table S2).

## 4 Discussion and Conclusion

The human-adapted MTBC exhibits a phylogeographical evolutionary pattern, amongst which *Maf* samples display strong geographic association with the West-African inhabitants (Isea-Peña et al., 2012; Comín et al., 2021; Coscolla et al., 2021). Lineage identification is of considerable significance in tuberculosis control, as it helps in quick diagnosis leading to effective treatment and prevention of potential future outbreaks (Dou et al., 2017; Napier et al., 2020). Although extensive studies pertaining to the differentiation of L5 and L6 have been carried out in the past, recent reports revealed the existence of underlying sub-lineages (Ates et al., 2018; Coscolla et al., 2021; Sanoussi et al., 2021). Methods such as phylogeny and PCA have been used to understand the lineage distribution of L5 and L6, with reports of incongruous clustering of L5 (Coscolla et al., 2021). Recent studies have also suggested clustering of L5 using “RD711” and other large sequence polymorphisms (Comín et al., 2021; Coscolla et al., 2021, Sanoussi et al., 2021). Taking these observations into cognizance, an attempt was made to understand the fine-level population stratification of *Maf,* especially L5 and its sub-lineages using SNPs with the aid of model- and non-model-based clustering approaches.

Lineage-wise clustering was observed in the whole-genome phylogenetic tree with the three samples branching independently serving as ‘distinct intermediates’ between L5 and L6 clusters (Supplementary Figure S1). Owing to insufficient data for intermediate branches, these samples were excluded from further population genomics analysis. Model-based approaches such as STRUCTURE can be used when linkage disequilibrium is negligible in the data. In *Maf* samples, we found low linkage disequilibrium, which agrees with that reported for other MTBC isolates (Supply et al., 2003). Population stratification of *Maf* indicated two major clusters, corresponding to L5 and L6. Lineage L6 is known to be geographically restricted to West Africa, whereas L5 is known to move from West Africa to Central Africa (Coscolla et al., 2021). L6 samples clustered into three distinct sub-clusters in the phylogenetic tree, which hints at a well-differentiated genetic structure, as is also observed in earlier studies (Otchere et al., 2018; Coscolla et al., 2021). These three sub-clusters were found to be independently homogeneous populations with high *Fst* and moderate genetic diversity (within members of each sub-cluster), wherein only three samples were found to be admixed (Supplementary Tables S12, S13).

In case of L5, the clusters were not clearly resolved in the phylogenetic tree, as is evident from smaller branch lengths suggesting lower genetic differentiation. Based on the RD711 marker, L5 samples were distributed into two groups *viz.,* L5.1 and L5.2. The observed *Fst* between L5.1 and L5.2 was found to be lower than L6 sub-clusters (Supplementary Table S12). Relatively lower nucleotide diversity was observed in L5.1 sub-clusters when compared with that of L5.2 sub-clusters, which may be attributed to the fact that fewer samples were available for L5.2. It should be mentioned that genome-wide *Fst* is dependent on the number of samples studied.

The use of stringent criteria for membership assignment using STRUCTURE helped to identify admixed samples for delineation of clusters with high confidence. PCA and DAPC further support these observations wherein with reduced data dimensions, the *Maf* clustering remained the same as observed in STRUCTURE and phylogeny analysis. The clusters thus identified were used to derive “*total/core-cluster-specific-SNPs*” by filtering admixed samples. SNPs belonging to genes involved in drug resistance accounted for ∼1% of the total SNP set in both lineages. They were retained as the prevalence of drug resistance in *Maf* is very rare (Asante-Poku et al., 2015; Acquah et al., 2021). The “*core-sub-cluster-specific*” SNPs were derived by taking into account the “*total-cluster-specific-SNPs*”. This strategy ensured the identification of exclusive SNPs for each (sub)-cluster (Figure 2). Furthermore, the lineage-specific (L5 and L6) synonymous SNPs identified in our study were found to be in complete agreement with the specific biomarker SNPs reported in earlier studies (Napier et al., 2020). The occurrence of different *“core-cluster-specific*” SNPs in the same gene across different (sub)lineages hints at the association of these SNPs towards selective advantage or (host)-adaptation (Ofori-Anyinam et al., 2020). Few missense SNPs that are part of “*core-cluster-specific*” SNP data for L5 and L6 (sub)lineages (obtained in our study) corroborate with the deleterious mutations observed in genes part of central carbon metabolism and electron transport chain. This provides clues to the existing metabolic differences in *Maf* (Ofori-Anyinam et al., 2020). These missense SNPs hint towards the slow growth in L5 which may be due to their possible role in impairing energy metabolism and its related pathways. For instance, both *pca* and *mdh* genes carry missense mutations in L5, the production of oxaloacetate may hence get affected. It is interesting to note that “*core-cluster-specific*” SNPs belonging to genes part of the pentose phosphate pathway were found only in L6 and its sub-lineages. Hence, the proposed “*core-cluster-specific*” non-synonymous SNPs have the potential to be studied further to understand their specific roles in fitness, adaptation to specific ecological niches and growth.

The synonymous “*core-cluster-specific-SNPs*” were used for lineage assignment in the validation dataset, which revealed consistent performance both by including and excluding SNPs belonging to drug resistance–associated genes (Table 3, Supplementary Tables S14, S15). This observation supports the reports of sporadic occurrence of drug resistance in *Maf* (Acquah et al., 2021; Asante-Poku et al., 2015). The synonymous “*core-cluster-specific-SNPs*” were able to delineate previously unassigned 154 *Maf* samples (Supplementary Tables S1, S2). The absence of core-SNPs in the L5.2.3 sub-cluster may be due to higher π along with absence of monophyletic clustering. A total of seven samples belonging to L5.2.1 were recognized as previously identified sub-lineage L5.2 (Ates et al., 2018; Coscolla et al., 2021). However, two samples (SRA Acc ID: ERR2383622 and ERR2383618), previously described as NRC1 and 69, respectively (Ates et al., 2018), were found to group distinctly into a new sub-cluster, L5.2.2, in our study. Few *Maf* samples clustering with L5.2.3 and L5.2.4 in our study remained unassigned previously (Coscolla et al., 2021). Hence, this extensive analysis using different model-based and *de novo* methods aided to understand the population stratification within the L5 lineage. These (sub)lineage-specific SNPs can not only serve as biomarkers for rapid identification along with the previous barcodes developed for MTBC (Napier et al., 2020) but also enable further delineation of *Maf* (L5 and L6) sub-lineages. The “*core-cluster-specific-SNPs*,” when accompanied with appropriate functional experiments, promise to enhance our understanding of genotype–phenotype association.

This study provides an overview of the underlying genetic diversity of the *Maf* samples with additional emphasis on L5 sub-lineages. The methodology described has the potential to be extended to studies involving all MTBC lineages. Improved genetic diversity delineation of *Maf* is possible with the availability of additional *Maf* whole-genome samples and use of a suitable pan-genome or more closely related *Maf* genome as reference (instead of *M. tuberculosis* H37Rv). In conclusion, the identified cluster-specific SNPs can serve as markers and help in comprehending the “specialist” characteristics apart from understanding the evolutionary trajectory of MTBC.

## 5 Standard Biosecurity and Institutional Safety Procedures

The study only involves bioinformatics analysis of publicly available *M. africanum* samples, and hence standard biosafety and institutional safety procedures are not in the scope of the article.

## Data Availability

The original contributions presented in the study are included in the article/Supplementary Material, further inquiries can be directed to the corresponding author.

## Supplementary Material

Supplementary DataSheet S1 Phylogenetic tree of *Mycobacterium africanum* (D1 dataset).

Supplementary Figure S1 Phylogenetic tree of 383 *Mycobacterium africanum* samples.

Supplementary Figure S2 Population stratification of L5.1.1 & L5.2.

Supplementary Figure S3 Cumulative PCA plot of *Mycobacterium africanum* (D1 dataset).

Supplementary Figure S4 Cumulative PCA plot of *Mycobacterium africanum* L6 (D2 dataset).

Supplementary Figure S5 Cumulative PCA plot of *Mycobacterium africanum* L5.1 (Group1 dataset).

Supplementary Figure S6 Cumulative PCA plot of *Mycobacterium africanum* L5.2 (Group 2 dataset).

Supplementary Figure S7 DAPC scatter plot of *Mycobacterium africanum* (D1 dataset).

Supplementary Figure S8 Crossvalidation plot of *Mycobacterium africanum* (D1 dataset) using *xval* DAPC.

Supplementary Figure S9 DAPC scatter plot of *Mycobacterium africanum* L6 (D2 dataset).

Supplementary Figure S10 Crossvalidation plot of *Mycobacterium africanum* L6 (D2 dataset) using *xval* DAPC.

Supplementary Figure S11 Crossvalidation plot of *Mycoacterium africanum* L5.1 (Group 1 dataset) using *xval* DAPC.

Supplementary Figure S12 Crossvalidation plot of *Mycobacterium africanum* L5.2 (Group 2 dataset) using *xval* DAPC.

Supplementary Figure S13 DAPC scatter plot of *Mycobacterium africanum* L5.1 (Group 1 dataset).

Supplementary Figure S14 DAPC scatter plot of *Mycobacterium africanum* L5.2 (Group 2 dataset).

Supplementary Figure S15 Comparison of *L6-core-cluster-specific* SNPs with the existing literature.

Supplementary Figure S16 Comparison of *L5-core-cluster-specific* SNPs with the existing literature.

Supplementary Table S1 Lineage information and SRA accession details of *Mycobacterium africanum* samples used for understanding genetic diversity.

Supplementary Table S2 Mapping of lineage for *Mycobacterium africanum* samples used for validation of identified *core-cluster-specific*-SNPs.

Supplementary Table S3 Population stratification of L5 and L6 samples of *M. africanum* at *k* = 2.

Supplementary Table S4 Population stratification of L6 samples of *M. africanum* at *k* = 3.

Supplementary Figure S5 Population stratification of L5.1 samples of *M. africanum* at *k* = 2.

Supplementary Figure S6 Population stratification of L5.1 samples of *M. africanum* at *k* = 6.

Supplementary Figure S7 Population stratification of L5.2 samples of *M. africanum* at *k* = 4.

Supplementary Table S8 Comparison of lineage assignment for L5 (Group 1) samples with existing literature.

Supplementary Table S9 Comparison of lineage assignment for L5 (Group 2) samples with existing literature.

Supplementary Table S10 A‐C: Population stratification of *Mycobacterium africanum* (D1 dataset) using *de novo* clustering.

Supplementary Table S11 A‐C: Population stratification of *Mycobacterium africanum* L6 (D2 dataset) using *de novo* clustering.

Supplementary Table S12
*Fst* values of *Mycobacterium africanum* L6 and L5 sub-clusters.

Supplementary Table S13 Pi values of L5 and l6 (sub)clusters (obtained using STRUCTURE tool).

Supplementary Table S14 Detailed list of L6-cluster-specific SNPs.

Supplementary Table S15 Detailed list of L5-cluster-specific SNPs.

Supplementary Table S16 Mapping of L6-core-cluster-specific SNPs with growth attenuation studies.

## Abbreviations

DAPC, discriminant analysis of principal components; *F*_*st*_, fixation index; L5, *Mycobacterium tuberculosis* var. *africanum* lineage 5; L6, *Mycobacterium tuberculosis* var. *africanum* lineage 6; *Maf*, *Mycobacterium tuberculosis* var. *africanum*; MIRU-VNTR, mycobacterial interspersed repetitive units-variable number of tandem repeats (MIRU-VNTR) spoligotyping; MTBC, *Mycobacterium tuberculosis* complex; PCA, principal component analysis; PCR, polymerase chain reaction; RDs, regions of difference; RFLP, restriction fragment length polymorphism; SNP, single nucleotide polymorphism; SRA, Sequence Read Archive; TB, tuberculosis; WHO, World Health Organization.
