# Supplementary figures and images for: Understanding the Genetic Diversity of Mycobacterium africanum Using Phylogenetics and Population Genomics Approaches
Source: Front Genet. 2022 Apr 13;13:800083. doi: 10.3389/fgene.2022.800083 (PMC9043288; doi:10.3389/fgene.2022.800083)

File scale: 0.1

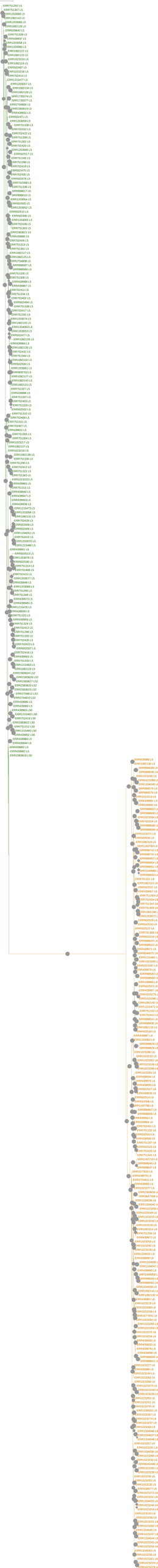

Supplement: Supplementary file 1 [file DataSheet1.ZIP › supplementary_fig/Supplementary DataSheet 1.pdf]

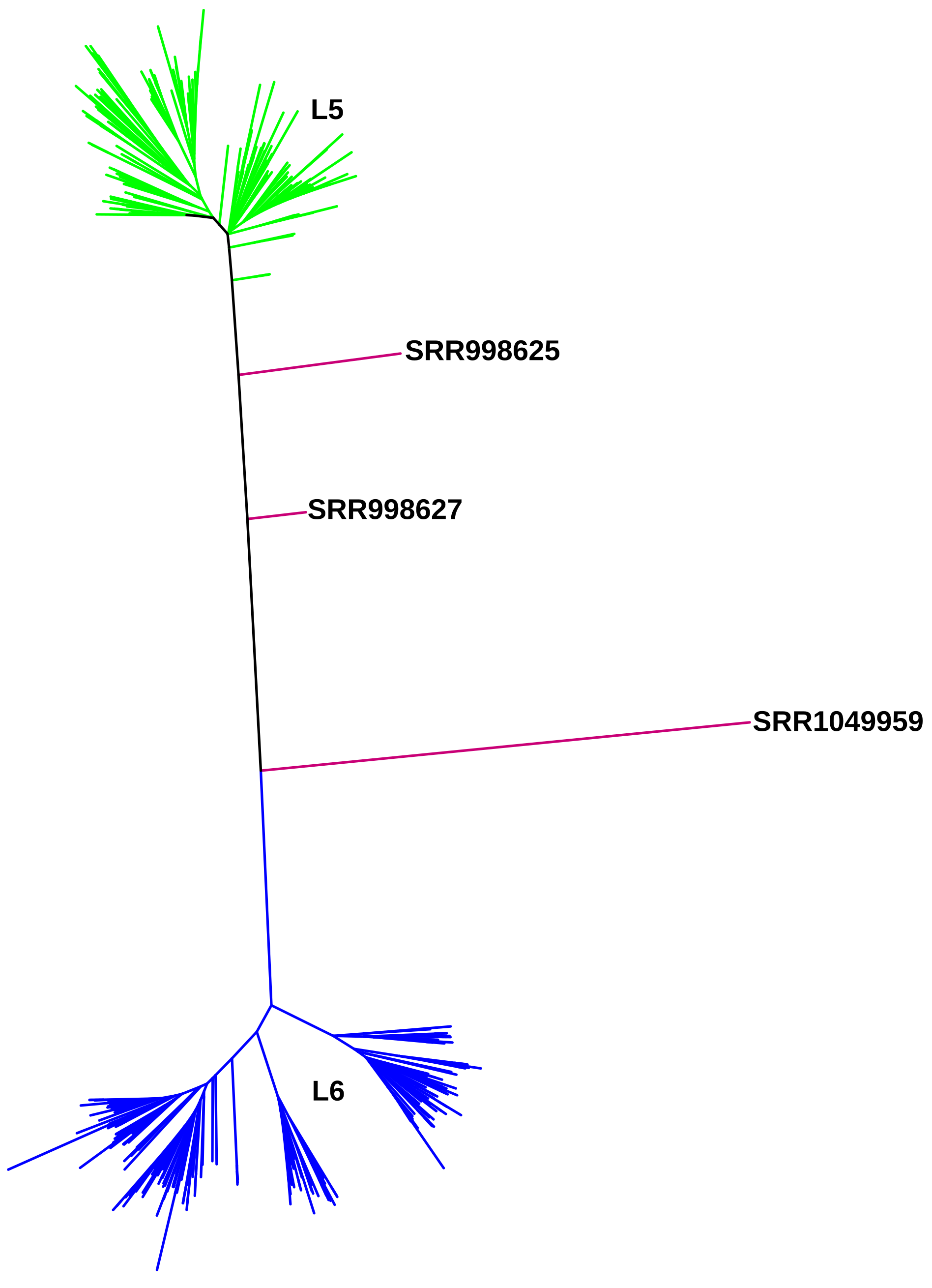

Supplement: Supplementary file 1 [file DataSheet1.ZIP › supplementary_fig/Supplementary_figure_S1.png]

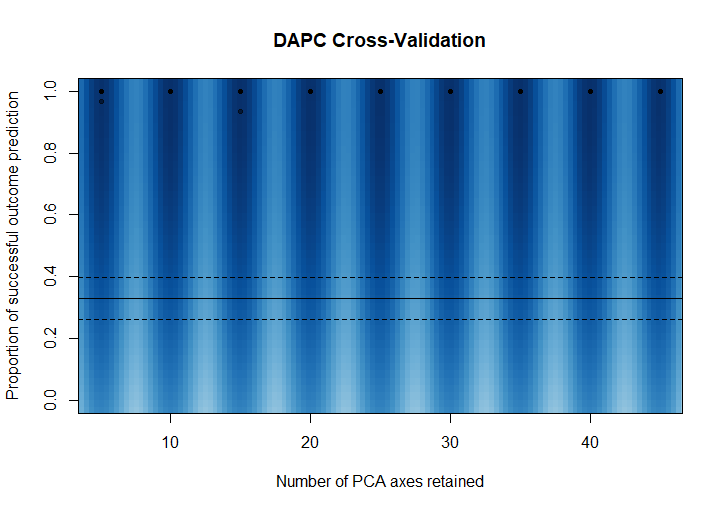

Supplement: Supplementary file 1 [file DataSheet1.ZIP › supplementary_fig/Supplementary_figure_S10.png]

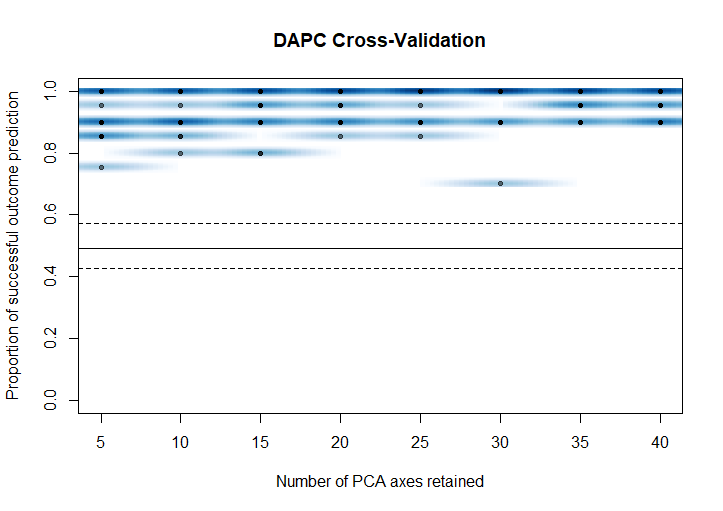

Supplement: Supplementary file 1 [file DataSheet1.ZIP › supplementary_fig/Supplementary_figure_S11.png]

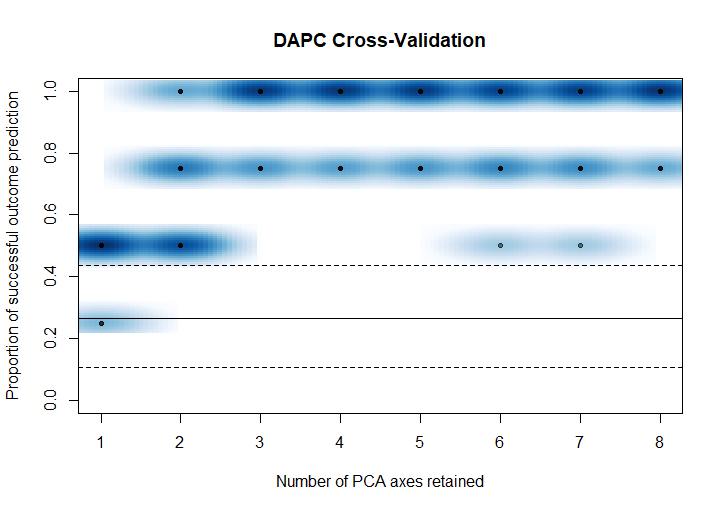

Supplement: Supplementary file 1 [file DataSheet1.ZIP › supplementary_fig/Supplementary_figure_S12.png]

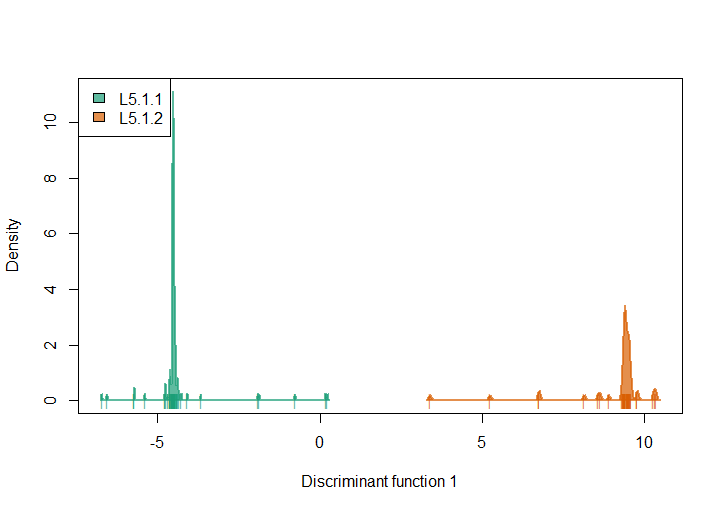

Supplement: Supplementary file 1 [file DataSheet1.ZIP › supplementary_fig/Supplementary_figure_S13.png]

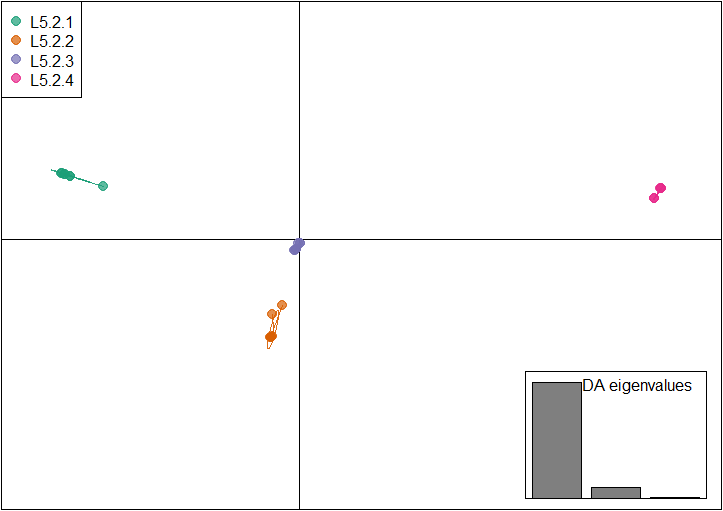

Supplement: Supplementary file 1 [file DataSheet1.ZIP › supplementary_fig/Supplementary_figure_S14.png]

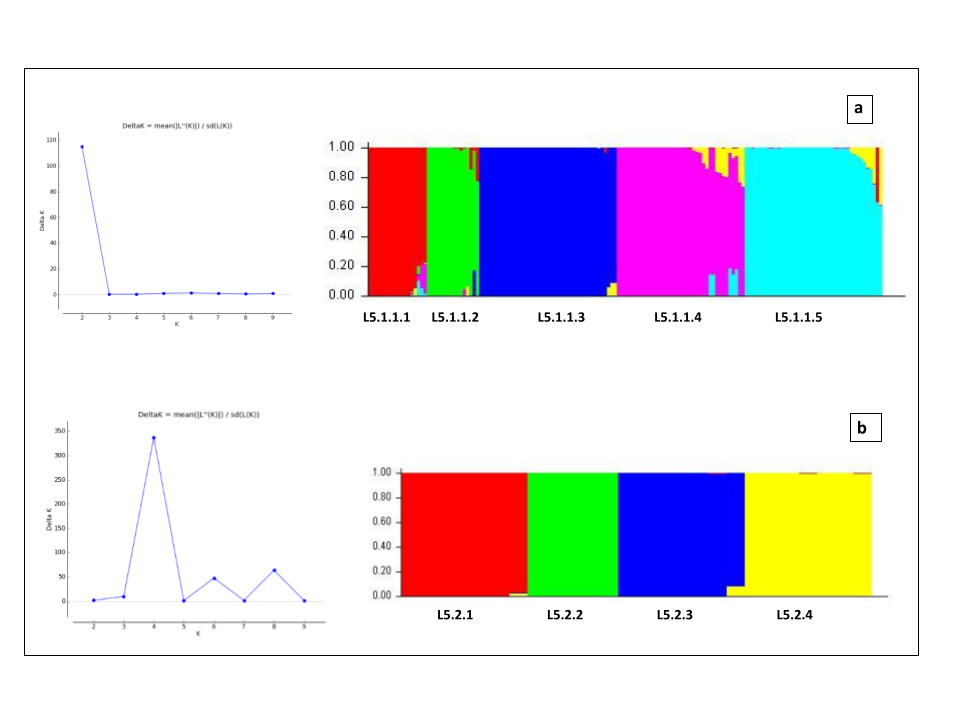

Supplement: Supplementary file 1 [file DataSheet1.ZIP › supplementary_fig/Supplementary_Figure_S2a-b.png]

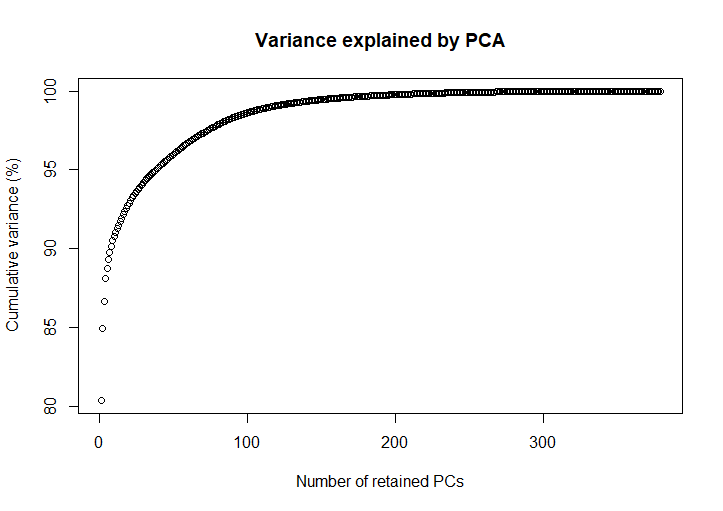

Supplement: Supplementary file 1 [file DataSheet1.ZIP › supplementary_fig/Supplementary_figure_S3.png]

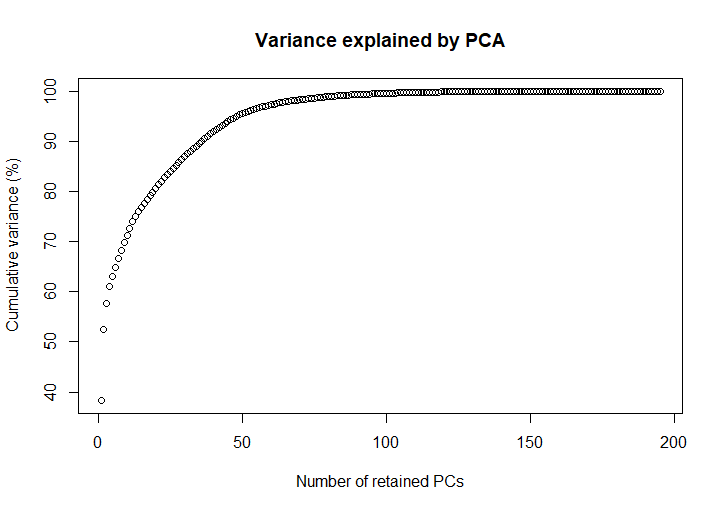

Supplement: Supplementary file 1 [file DataSheet1.ZIP › supplementary_fig/Supplementary_figure_S4.png]

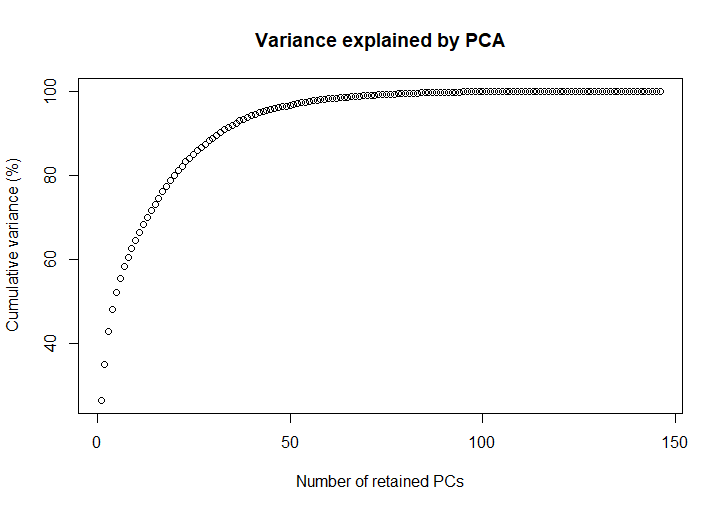

Supplement: Supplementary file 1 [file DataSheet1.ZIP › supplementary_fig/Supplementary_figure_S5.png]

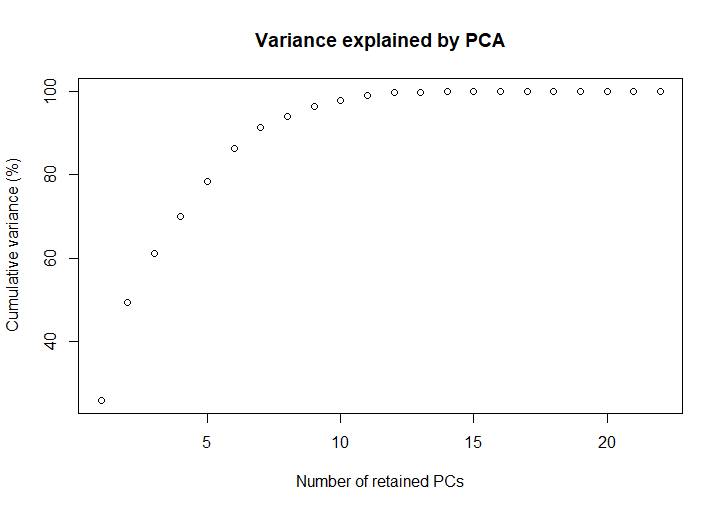

Supplement: Supplementary file 1 [file DataSheet1.ZIP › supplementary_fig/Supplementary_figure_S6.png]

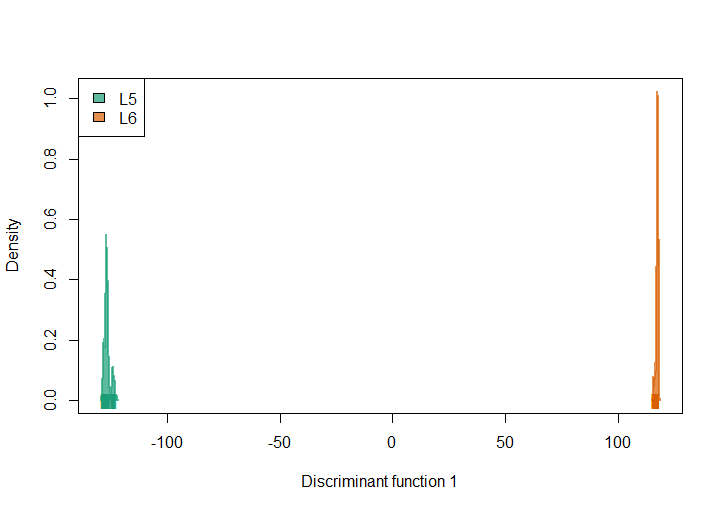

Supplement: Supplementary file 1 [file DataSheet1.ZIP › supplementary_fig/Supplementary_figure_S7.png]

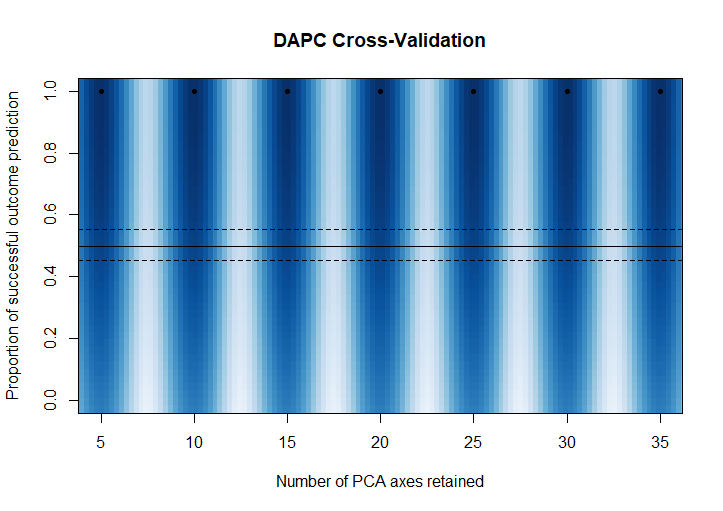

Supplement: Supplementary file 1 [file DataSheet1.ZIP › supplementary_fig/Supplementary_figure_S8.png]

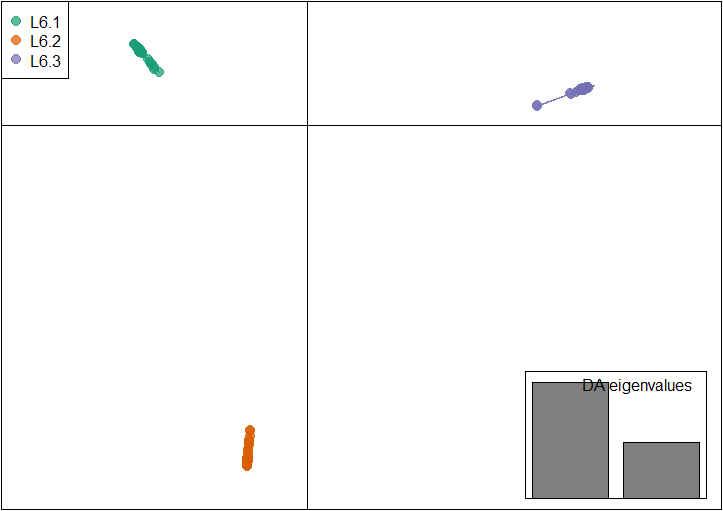

Supplement: Supplementary file 1 [file DataSheet1.ZIP › supplementary_fig/Supplementary_figure_S9.png]

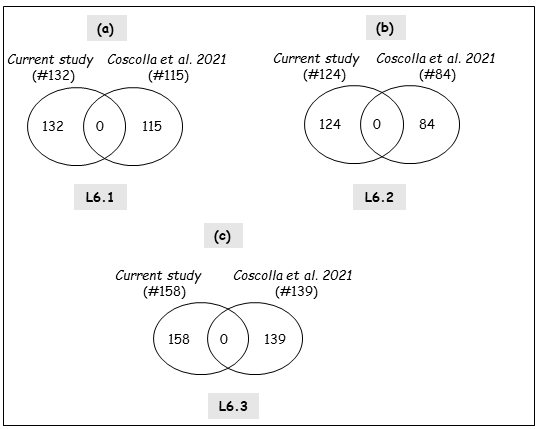

Supplement: Supplementary file 1 [file DataSheet1.ZIP › supplementary_fig/Suuplementary_figure_S15.png]

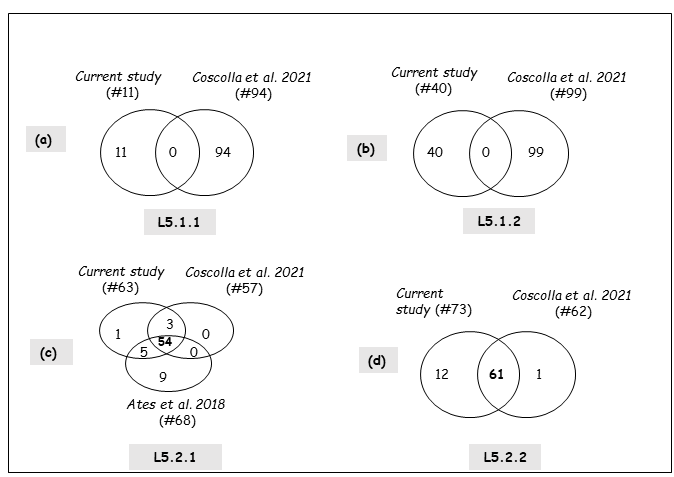

Supplement: Supplementary file 1 [file DataSheet1.ZIP › supplementary_fig/Suuplementary_figure_S16.png]
