# Supplementary material for: Understanding the Genetic Diversity of Mycobacterium africanum Using Phylogenetics and Population Genomics Approaches
Source: Front Genet. 2022 Apr 13;13:800083. doi: 10.3389/fgene.2022.800083 (PMC9043288; doi:10.3389/fgene.2022.800083)
Supplement: Supplementary file 2 [file DataSheet2.ZIP › supplementary_tables/Supplementary_Table_S10.docx]

**Supplementary Table S10**: Population Stratification of *Mycobacterium africanum* (D1 dataset) using *de novo*  clustering


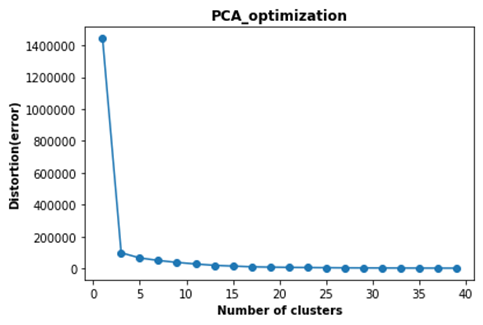


**Supplementary Table S10a**: Population stratification of *Mycobacterium africanum* (D1 dataset) using *de novo* (K-means) clustering


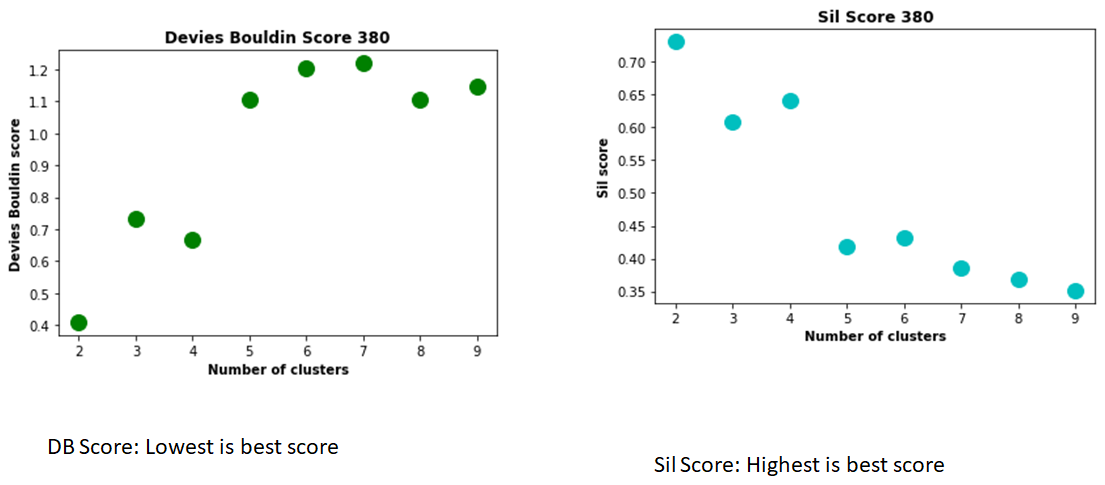


**DB Score**

**Supplementary Table S10 b**: Population stratification of *Mycobacterium africanum* (D1 dataset) using *de novo* (Davies Bouldin Score) clustering


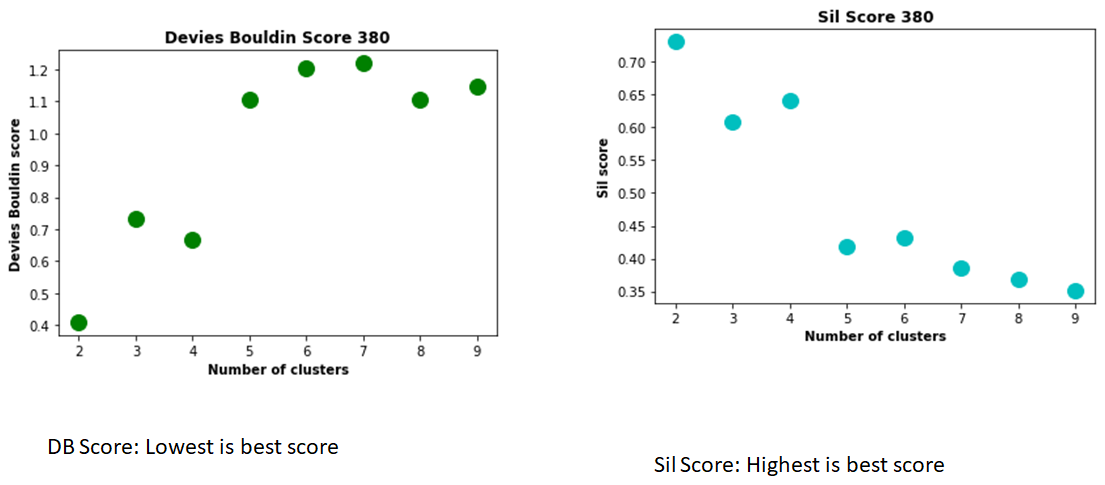


**Supplementary Table S10 c**: Population stratification of *Mycobacterium africanum* (D1 dataset) using *de novo* (Silhouette Score) clustering
