# Supplementary material for: Understanding the Genetic Diversity of Mycobacterium africanum Using Phylogenetics and Population Genomics Approaches
Source: Front Genet. 2022 Apr 13;13:800083. doi: 10.3389/fgene.2022.800083 (PMC9043288; doi:10.3389/fgene.2022.800083)
Supplement: Supplementary file 2 [file DataSheet2.ZIP › supplementary_tables/Supplementary_Table_S11.docx]

**Supplementary Table S11:** Population stratification of *Mycobacterium africanum* L6 (D2 dataset) using *de novo* clustering


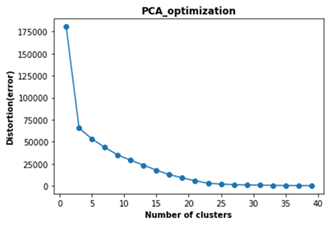


**Supplementary Table S11a** : Population stratification of *Mycobacterium africanum* L6 (D2 dataset) using *de novo* (K-means) clustering


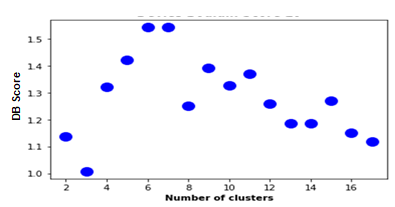


**Supplementary Table S11b** : Population stratification of Mycobacterium africanum L6 (D2 dataset) using de novo (Davies Bouldin Score) clustering


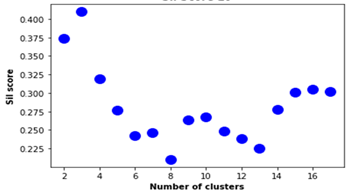


**Supplementary Table S11c** : Population stratification of Mycobacterium africanum L6 (D2 dataset) using de novo (Silhouette Score) clustering
